# Electronic tuning of tetranuclear Cu(i)–pyrazolate macrocycles: anti-Kasha emission through high-lying triplet states of a C_2_F_5_-substituted complex

**DOI:** 10.1039/d6dt01868h

**Published:** 2026-10-13

**Authors:** Shinaj K. Rajagopal, Christian M. Peterson, Matthias Zeller, Sergei Savikhin, Lyudmila V. Slipchenko, Alexander Wei

**Affiliations:** a James and Margaret Tarpo Department of Chemistry, Purdue University 560 Oval Drive West Lafayette IN 47907 USA alexwei@purdue.edu; b Department of Physics and Astronomy, Purdue University 525 Northwestern Ave West Lafayette IN 47907 USA; c Department of Materials Engineering, Purdue University, Neil Armstrong Hall of Engineering 701 W Stadium Ave West Lafayette IN 47907 USA

## Abstract

The phosphorescence of tetranuclear Cu(i)–pyrazolate macrocycles (Cu_4_(R-pz)_4_; pz = 3,5-di-^*t*^Bu-pyrazolyl) can be influenced by electronic as well as steric factors of the R group at C4, however separating their effects can be challenging. This issue has been addressed in the study of a Cu(i)–pyrazolate tetramer with C4 pentafluoroethyl groups (Cu_4_(C_2_F_5_-pz)_4_1), a homolog of Cu_4_(CF_3_-pz)_4_2. C_2_F_5_-pz was synthesized by a novel route and combined with CuPF_6_ to form macrocycle 1; X-ray crystal analysis at 150 K revealed all peripheral C_2_F_5_ groups adopt *endo* conformations and impart similar torsional strain as the CF_3_ groups in 2. The C_*x*_F_*y*_ units have a strong inductive effect on the luminescent behavior of thin-film samples. Excitation of 1 at 270 nm (∼4.6 eV) produces bimodal emission with peaks centred at 450 and 520 nm and a marked increase in intensity of the higher-energy (HE) band at low temperatures, whereas 2 exhibits the opposite trend. TD-DFT analysis of Cu_4_(C_2_F_5_-pz)_4_1 reveals cluster-centred (CC) orbitals mixed with MLCT character for most excited states, with spin–orbit coupling (SOC) and singlet–triplet energy gap (Δ*E*_ST_) analysis indicating favourable intersystem crossing (ISC) between S_1_ and high-lying T_*n*_ states. The dual HE/LE emission is ascribed to a large energy gap between high- and low-lying states within the triplet manifold.

## Abstract 2

The inductive effect of C_2_F_5_ on the photoluminescence of copper–pyrazolate tetramers results in dual emission at 450 and 520 nm. Blue emission is produced from high-lying excited states with hybrid ^3^CC and ^3^MLCT character.

## Graphical abstract

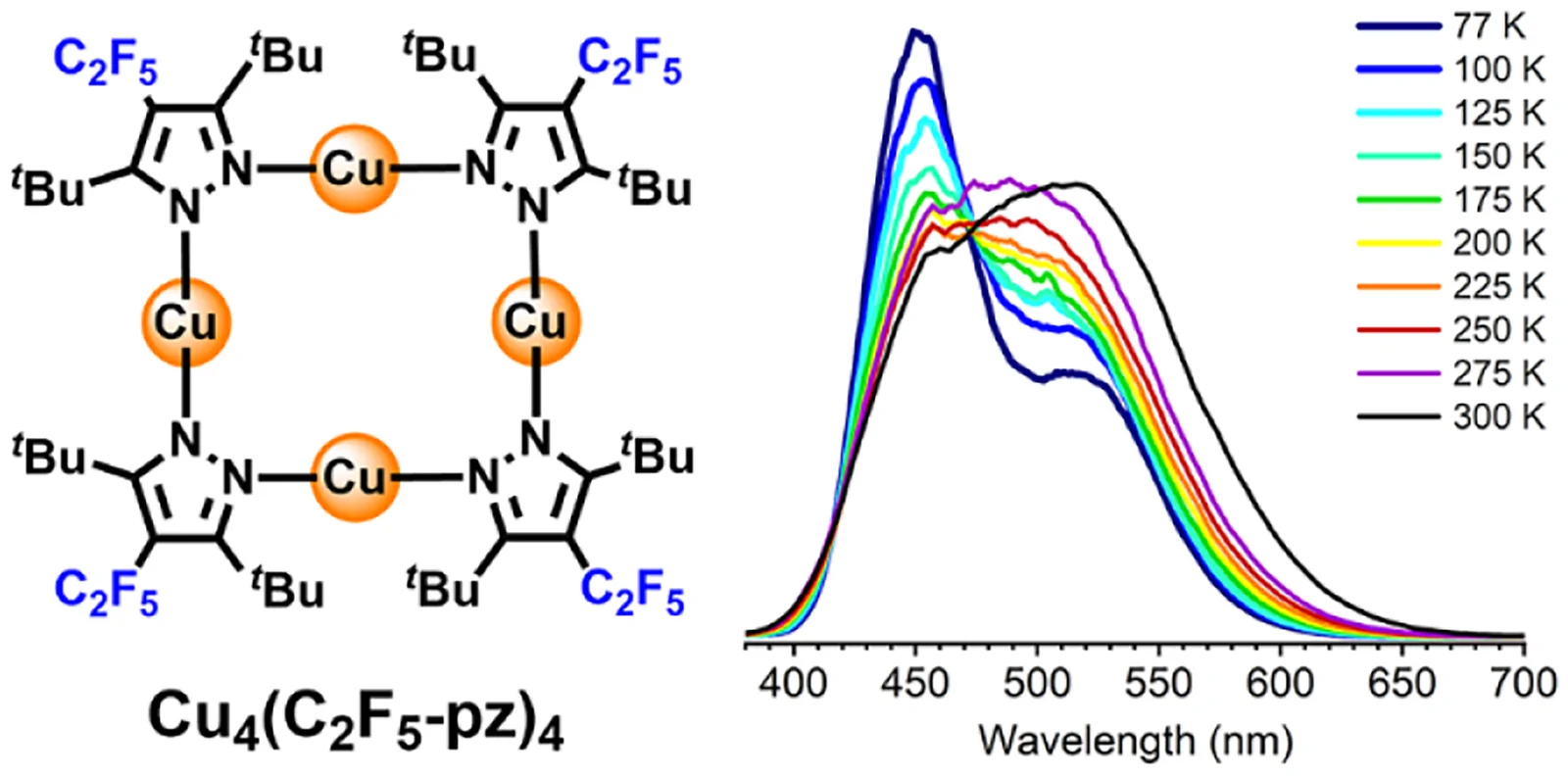

## Introduction

There is strong interest in luminescent Cu(i) complexes as long-lived, inexpensive phosphors for solid-state lighting.^1,2^ Organic light-emitting diodes (OLED) and phosphorescent OLEDs (PHOLEDs) are prime candidates for next-generation displays, with the latter being capable of providing 100% internal quantum efficiency.^3^ Blue PHOLEDs face technical hurdles however, as higher excitation energies needed to produce blue light can accelerate the degradation of organic phosphors. The relative stability of Cu-coordination complexes and their ease of handling thus make them serious contenders as practical blue PHOLEDs.^4–6^

We have been investigating the photoluminescent (PL) properties of Cu(i)–pyrazolate complexes that form macrocyclic tetramers (Cu_4_(R-pz)_4_, where pz = 3,5-di-^*t*^Bu-pyrazolyl and R = group at C4; Fig. 1).^7–9^ These are homologues of the more widely studied Cu(i)–pyrazolate trimers (Cu_3_pz_3_), whose diverse structure–PL relationships can support numerous optical and optoelectronic applications including PHOLEDs, anti-counterfeit measures, and sensors.^10–12^ PL studies of Cu_4_pz_4_ complexes are relatively few,^7,8,13–15^ but their potential for such applications are just as exciting. One important distinction is that Cu_4_pz_4_ complexes are molecular emitters whose solid-state PL have low sensitivity to packing density, enabling their tuneable loading into host matrix materials,^7^ whereas emissions from Cu_3_pz_3_ complexes are highly sensitive to supramolecular associations.^10,16,17^ Several Cu_4_pz_4_ complexes are able to produce room-temperature phosphorescence (RTP) at deep-blue wavelengths (455–460 nm) with high quantum yield,^7,9^ which is of great interest to PHOLED design.^18,19^ The high-energy emissions have been ascribed to (i) facile HOMO–LUMO transitions between cluster-centred (CC) states, and (ii) macrocyclic conformations which position the Cu atoms into tightly packed cores that resist excited-state reorganization, a novel manifestation of rigidochromism.^7^ The Cu–Cu distances and geometry of the Cu_4_ cluster are highly sensitive to long-range (transannular) steric interactions that are driven by local steric effects of the R groups on flanking *^t^*Bu units.^7–9^

**Fig. 1:**
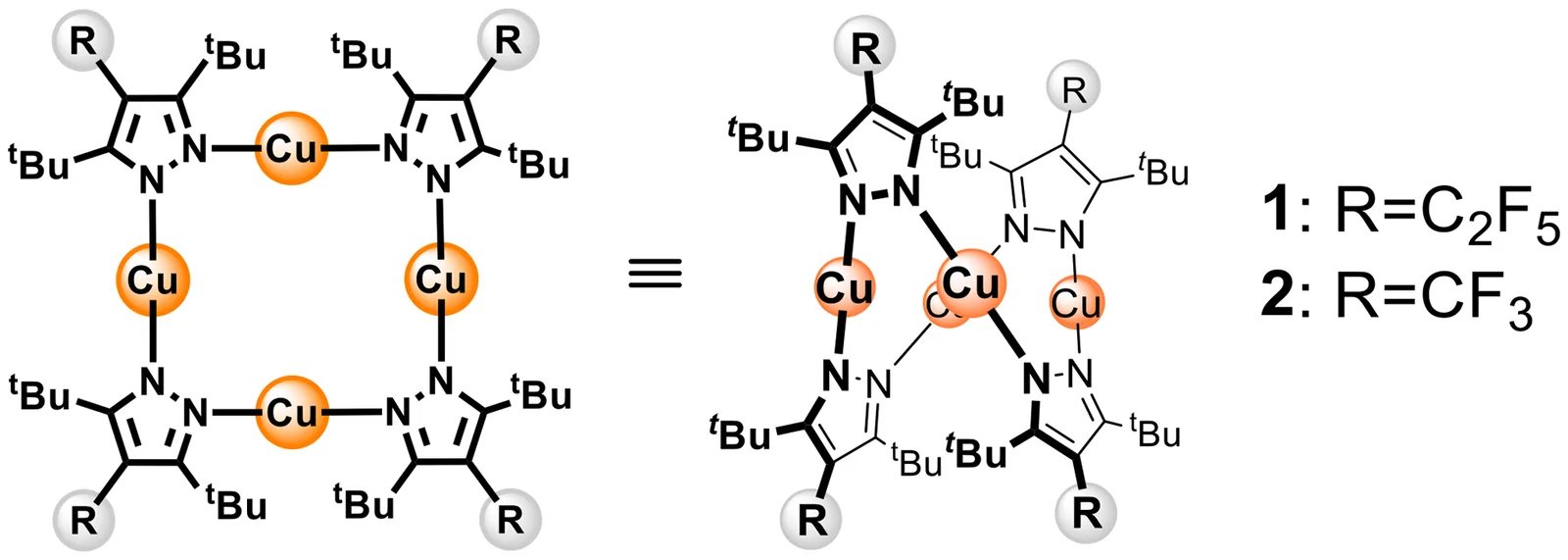
Planar view and saddle-shaped conformation of Cu_4_(R-pz)_4_ macrocycle (pz = 3,5-di-*^t^*Bu pyrazolyl; R = C4 substituent).

Emission wavelengths can be influenced by electronic as well as steric or conformational effects, most often through changes in excited-state T_1_ energy. However, Cu_4_pz_4_ species have complex structures that hinder simple correlations between electronic parameters and emission energies. For example, a Cu_4_pz_4_ complex with electron-deficient ligands (R = CF_3_; 2) emits broadly across blue and green wavelengths whereas one with electron-rich ligands (R = CH_3_) emits mainly in the blue; the difference is due more to increased steric crowding imposed by CF_3_ than to its electronegativity.^8^ The use of time-dependent density functional theory (TD-DFT) on a case-by-case basis is perhaps the best approach for elucidating the relative impact of electronic density on PL.

Here we present a Cu_4_pz_4_ derivative (R = C_2_F_5_; 1) whose C4 groups impose similar steric and electronic influence as Cu_4_(CF_3_-pz)_4_2, but with substantial differences in photophysical behaviour. The solid-state PL of Cu_4_(C_2_F_5_-pz)_4_1 produces more blue emission than 2, and variable-temperature (VT) PL analysis reveals dual emission peaks centred at 450 and 520 nm, providing clear evidence of a thermosensitive electronic effect. TD-DFT calculations show that the excited states of 1 possess mixed CC and metal–ligand charge transfer (MLCT) character, the latter being enhanced by the electronegative C_2_F_5_ units. Spin–orbit coupling (SOC) is likely mediated through high-lying triplet states (T_*n*_; *n* > 1), whose roles in photoemission have been examined previously for Cu_3_pz_3_ complexes^16,20,21^ and other organic phosphors.^22–24^ However, Cu_4_(C_2_F_5_-pz)_4_1 is unique in its strong reliance on ^3^CC states to produce deep-blue emission, and in a manner distinct from other Cu_4_pz_4_ complexes.^7,9^

## Results and discussion

### Synthesis

C_2_F_5_-substituted pyrazole 3 was synthesized from *N*-benzyl-3,5-di-*tert*-butyl-4-iodopyrazole^8^ in two steps and 53% overall yield (Scheme 1). Nucleophilic addition of the C_2_F_5_ group involved *in situ* formation of CuC_2_F_5_ from TMS-CF_3_ (Ruppert–Prakash reagent)^25^ followed by Pd-catalyzed hydrogenation. C_2_F_5_-pz 3, Cu(CH_3_CN)_4_PF_6_, and Et_3_N were combined in MeOH to form Cu_4_(C_2_F_5_-pz)_4_1, which was collected as a precipitate in 87% yield and purified by sublimation.

**Scheme 1:**
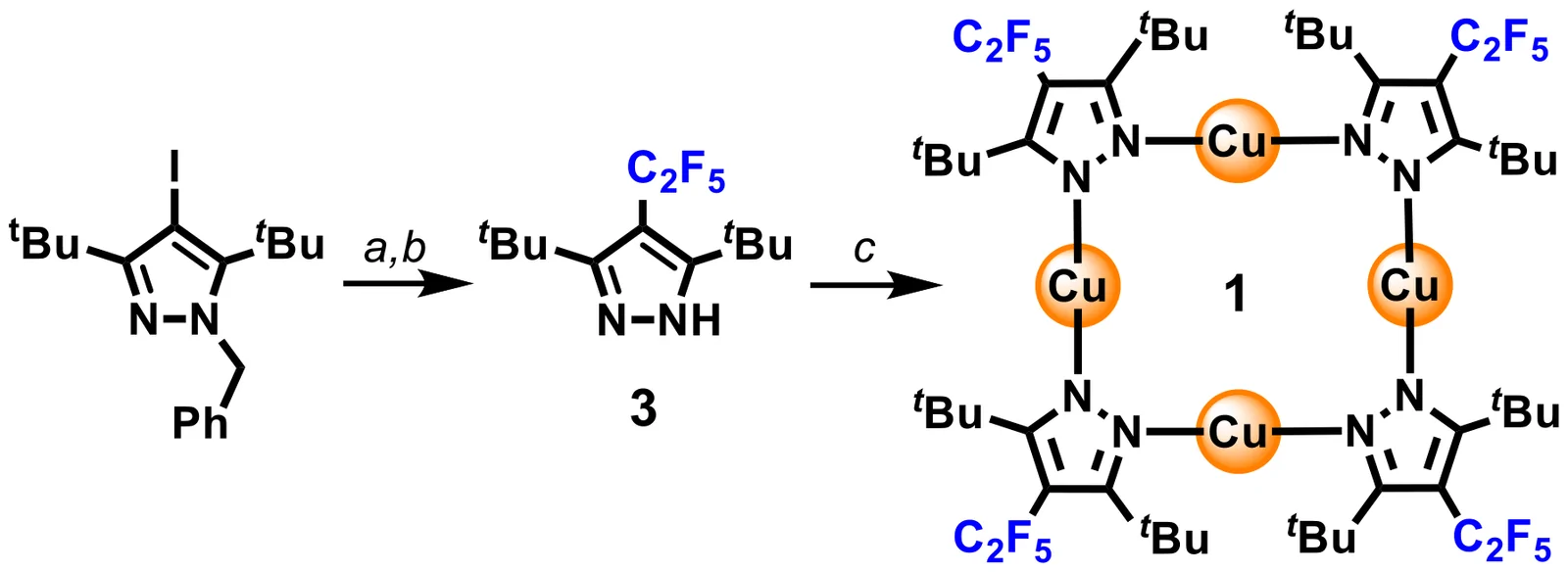
Synthesis of Cu_4_(C_2_F_5_-pz)_4_1. (a) TMSCF_3_, CuBr, KF, DMF, 60 °C. (b) 5% Pd/C (cat.), H_2_ (1 atm), 1 : 1 EtOAc : MeOH, rt. (c) [Cu(CH_3_CN)_4_]PF_6_ (1 eq.), Et_3_N (1 eq.), MeOH, rt.

### X-ray crystallography

Single crystals grown from a toluene/CH_2_Cl_2_ solution of 1 were submitted to X-ray analysis, which confirms that the macrocycle adopts a saddle-shaped conformation similar to that of other Cu_4_pz_4_ species and aza[12-Cu-4] metallacrowns.^26^ Analysis at 150 K yields an orthorhombic unit cell with all C_2_F_5_ groups rotated *endo* relative to the macrocycle (Fig. 2a). The all-*endo* geometry of 1 yields a prolate shape that maximizes crystal packing, although the void space within the unit cell (19.6%) is still rather large (Fig. 2b). The bulky C4 substituents also influence the local and global conformational behaviour of 1: the C_2_F_5_ units are tightly wedged between flanking ^*t*^Bu groups with a mean nearest-neighbour F⋯H distance of 2.35 Å, well below the sum of their vdW radii (2.6–2.7 Å; Fig. 2c).^27^ Further, the ^*t*^Bu groups are displaced from bisected conformations which increases transannular steric interactions (Fig. 2d), causing the pyrazolate ligands to swivel.^9,28^ The steric crowding forces the Cu atoms of 1 to adopt a nonplanar quadrangle with an aspect ratio (*d*_L_/*d*_S_) of 1.03 (Fig. 2e), similar to that observed in the X-ray analysis of Cu_4_(CF_3_-pz)_4_2.^8^

**Fig. 2:**
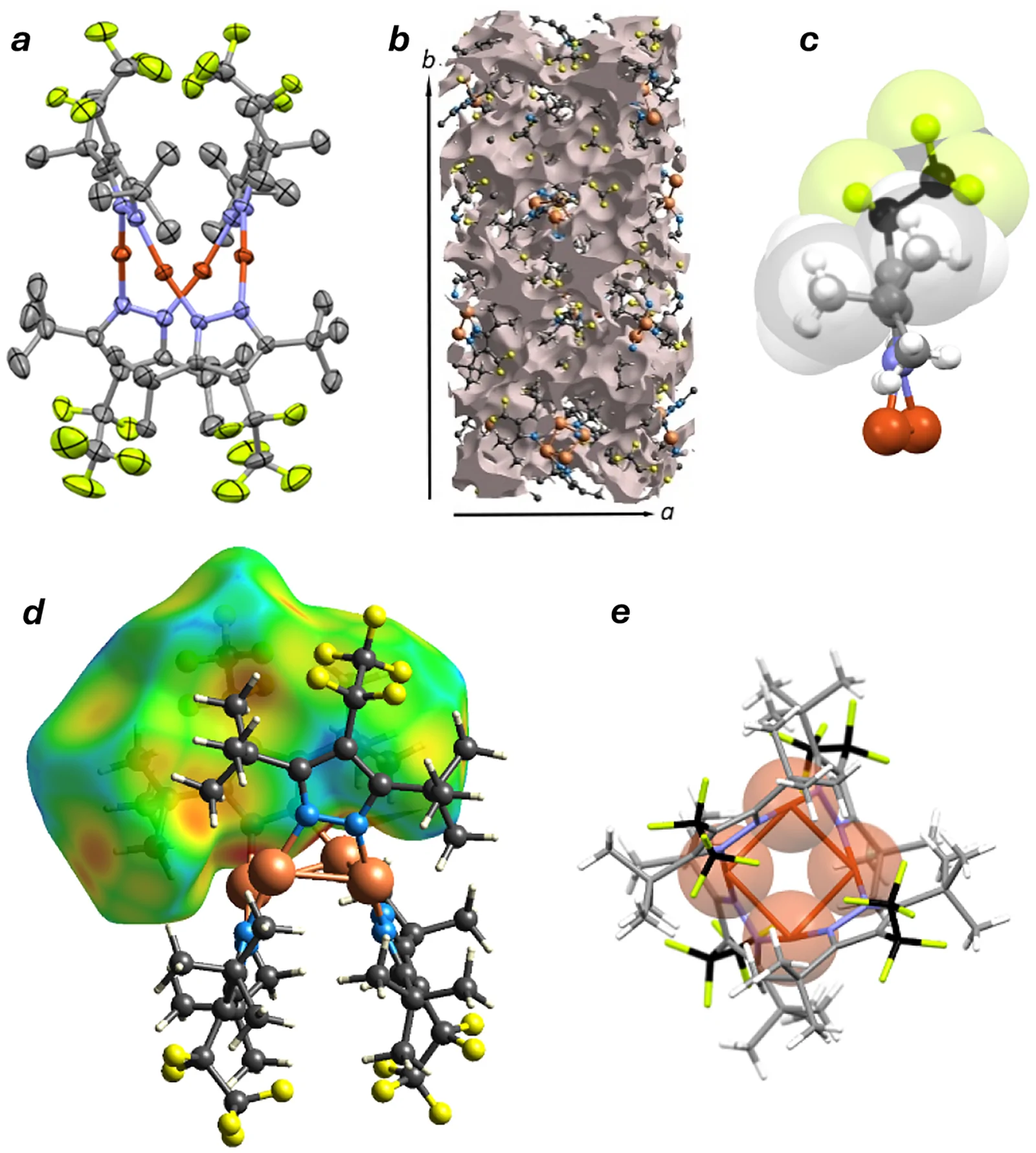
(a) X-ray crystal structure of 1 at 150 K; thermal ellipsoids drawn at the 50% probability level with H atoms removed for clarity. (b) Hirshfeld surface analysis of void volume (19.6%) in orthorhombic unit cell (*a* = 19.582, *b* = 42.415, *c* = 14.881 Å). (c) Edge view of *endo*-C_2_F_5_-pz ligand in 1 with flanking ^*t*^Bu groups offset from ideal bisected conformation. (d) Side view of 1 with isosurface showing regions of close contact between opposing C_2_F_5_-pz ligands. (e) Plan view of 1 with Cu_4_ atoms in spacefill mode.

Despite some similarities, the conformations of crystalline Cu_4_(C_2_F_5_-pz)_4_1 and Cu_4_(CF_3_-pz)_4_2 differ both at the local and global level. Most ^*t*^Bu groups in 1 are canted with two methyl units projected *endo* (in the same direction as the C_2_F_5_ group), whereas those of 2 tend to have one *endo* methyl unit (Fig. 3). This difference has a strong impact on transannular steric interactions: the opposing pyrazolate ligands in 1 are oriented such that the *endo* C_2_F_5_ and ^*t*^Bu methyl groups are tightly packed with multiple short-range contacts (Fig. 2d), whereas some gaps exist between the *endo* surfaces of 2. Thus, it can be argued that 1 is conformationally more restricted than 2 in their crystalline states.

**Fig. 3:**
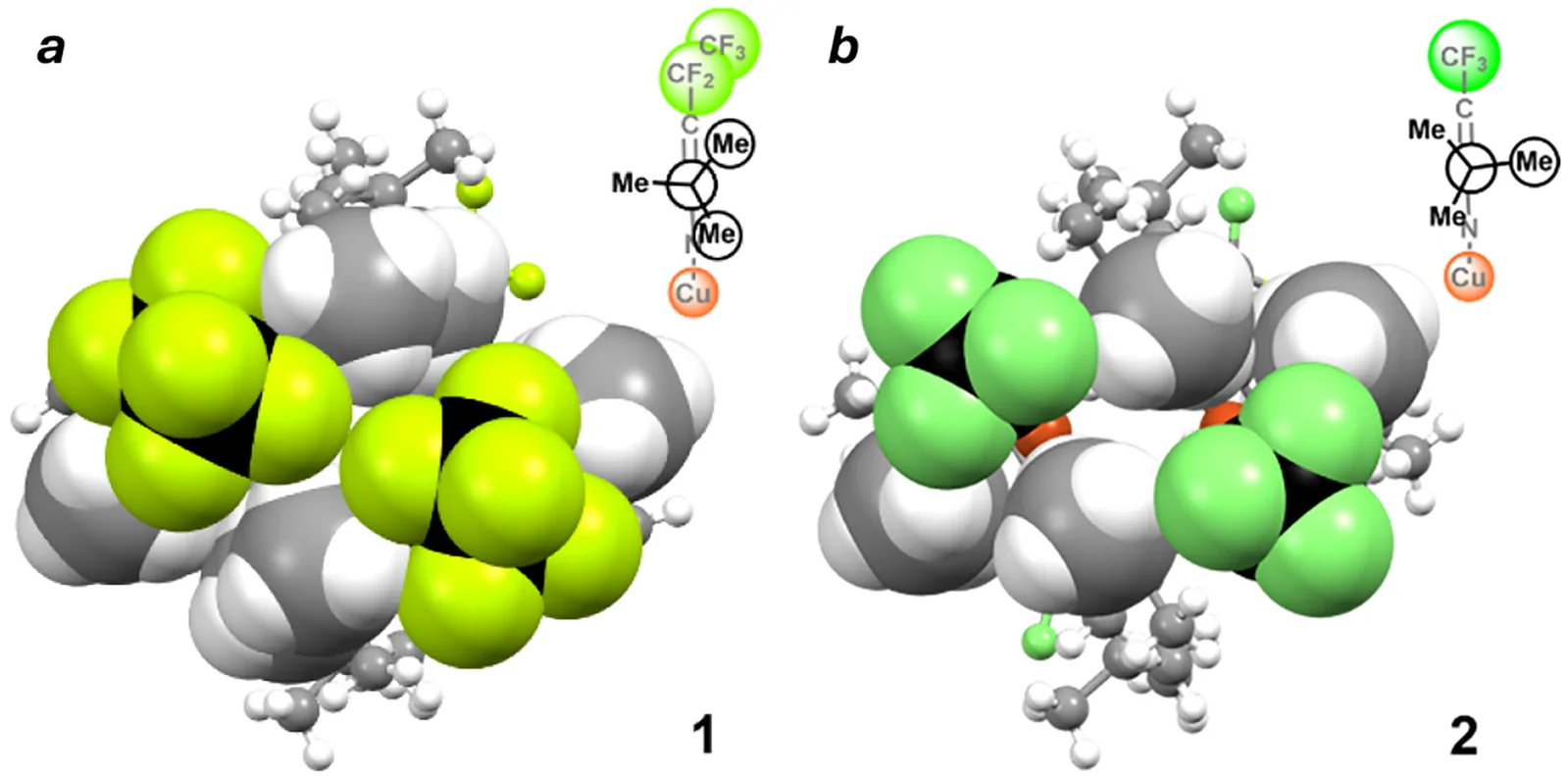
X-ray crystal structures (150 K) emphasizing transannular interactions between *endo* substituents (facing right in Newman projections). (a) Cu_4_(C_2_F_5_-pz)_4_1 with all C_2_F_5_ and methyl groups having at least one transannular contact. (b) Cu_4_(CF_3_-pz)_4_2 (conformer I)^8^ showing no transannular contacts for CF_3_ groups.

### Photoluminescence studies

Cu_4_(C_2_F_5_-pz)_4_1 in powder and thin-film form produce blue–green luminescence when irradiated with a UV lamp (Fig. 4a). PL analysis at 295 K indicates a broad, bimodal emission with a maximum at 520 nm and a sizeable shoulder centred at 450 nm (Fig. 4b). The PL quantum yield (*Φ*_PL_) and decay lifetime at 520 nm is 87% and 38.3 μs, indicating efficient RTP (Fig. S1, SI). All absorbance and excitation spectra of 1 (*λ*_em_ 450 or 520 nm) show a peak at 280 nm, which supports the primary role of CC states in RTP.^14^ We note that ^3^CC states are more commonly invoked in low-energy (LE) emissions from Cu(i) complexes,^6,29^ and examples of their participation in high-energy (HE) emissions remain rare.^7,9,28^

**Fig. 4:**
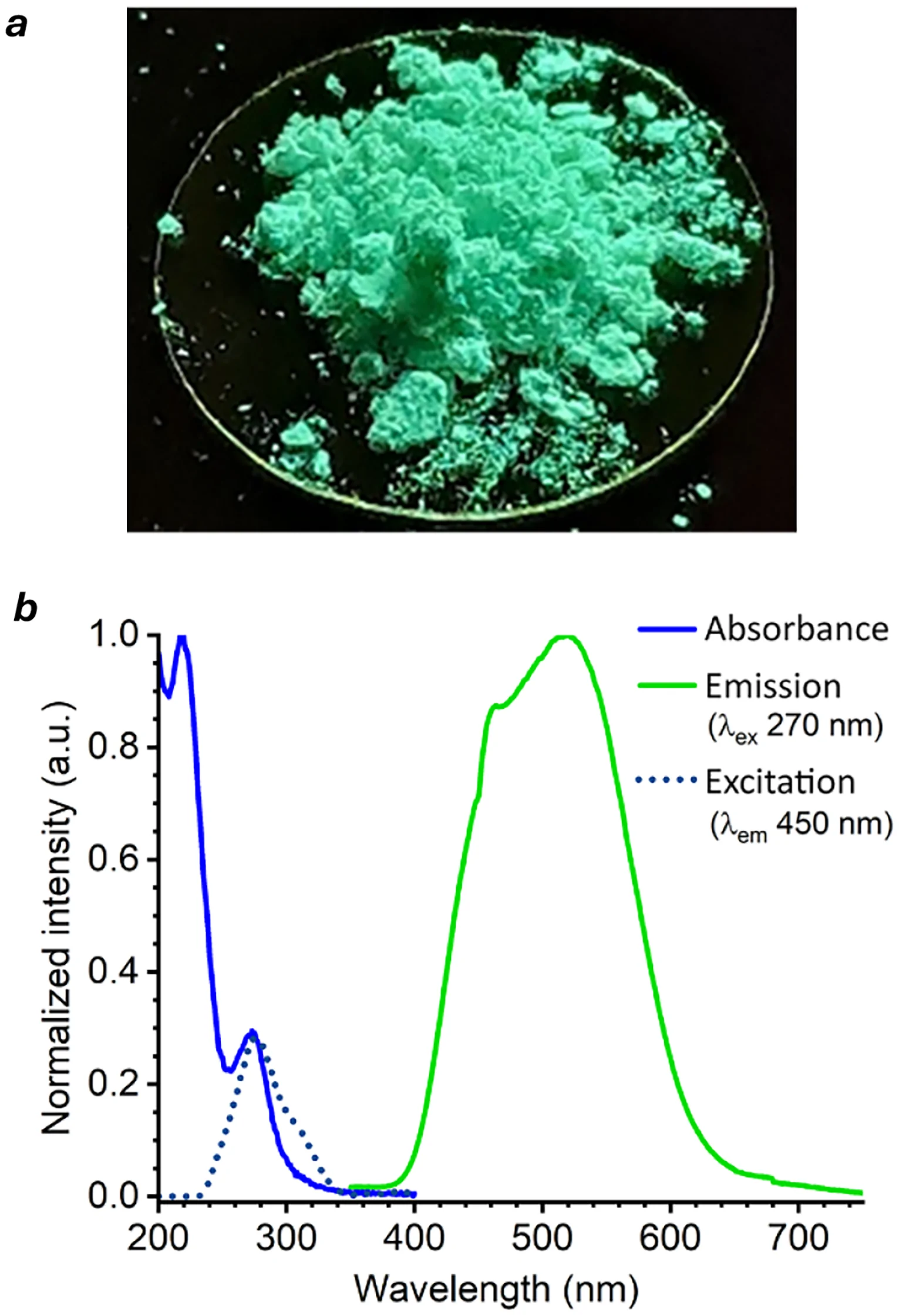
(a) Cu_4_(C_2_F_5_-pz)_4_1 as a luminescent powder (*λ*_ex_ 254 nm). (b) Absorbance, PL, and excitation spectra of 1 (thin film on quartz; *λ*_ex_ 270 nm) with bimodal emission at 450 nm (HE) and 520 nm (LE).

PL emission from Cu_4_pz_4_ complexes is mediated chiefly by the Cu_4_ core, whose geometry plays a critical role in *λ*_em_. Prior studies have shown that blue emission is produced if the Cu_4_ cluster adopts a planar, close-packed rhombus, whereas deviations from that geometry result in longer *λ*_em_ due to excited-state contraction.^7–9^ The LE emission band of 1 centred at 520 nm also correlates with a less densely packed Cu_4_ core, meaning that the conformational rigidity of 1 in its ground state is not sufficient to prevent changes in the excited-state geometry of the Cu_4_ cluster. However, the HE emission band at 450 nm is intriguing as the excitation spectrum is dominated by the CC state (Fig. 4b), suggesting an influence by factors beyond steric or conformational effects.

VT-PL studies performed on a thin film of 1 provide several important insights. First, lowering the temperature increases the definition and intensity of the HE band; the 450 nm peak is well resolved at or below 100 K and dominates the emission spectrum (Fig. 5a). An increase in blue emission can also be observed upon immersing a powder sample in liquid N_2_ (Movie S1, SI). Second, the VT-PL spectra form an isosbestic point close to 470 nm, indicative of a dual-emission profile with essentially no change in PLQY (Fig. S2, SI). This also means that ^3^CC emissions from 1 are not competing with non-radiative processes, enabling us to consider the two-state emission as a function of temperature. Third, comparison with a VT-PL analysis of Cu_4_(CF_3_-pz)_4_2 performed under identical conditions reveals opposite trends in temperature dependency for HE and LE emissions (Fig. 5b). Photoemission from solid-state Cu_4_pz_4_ complexes is unimolecular and precludes lattice structure as a contributing factor, so long as the molecules are fully immobilized.^7^ We note that 2 in its crystalline form undergoes a conformational transition at lower temperatures, although this does not affect the trend observed in VT-PL analysis.^8^

**Fig. 5:**
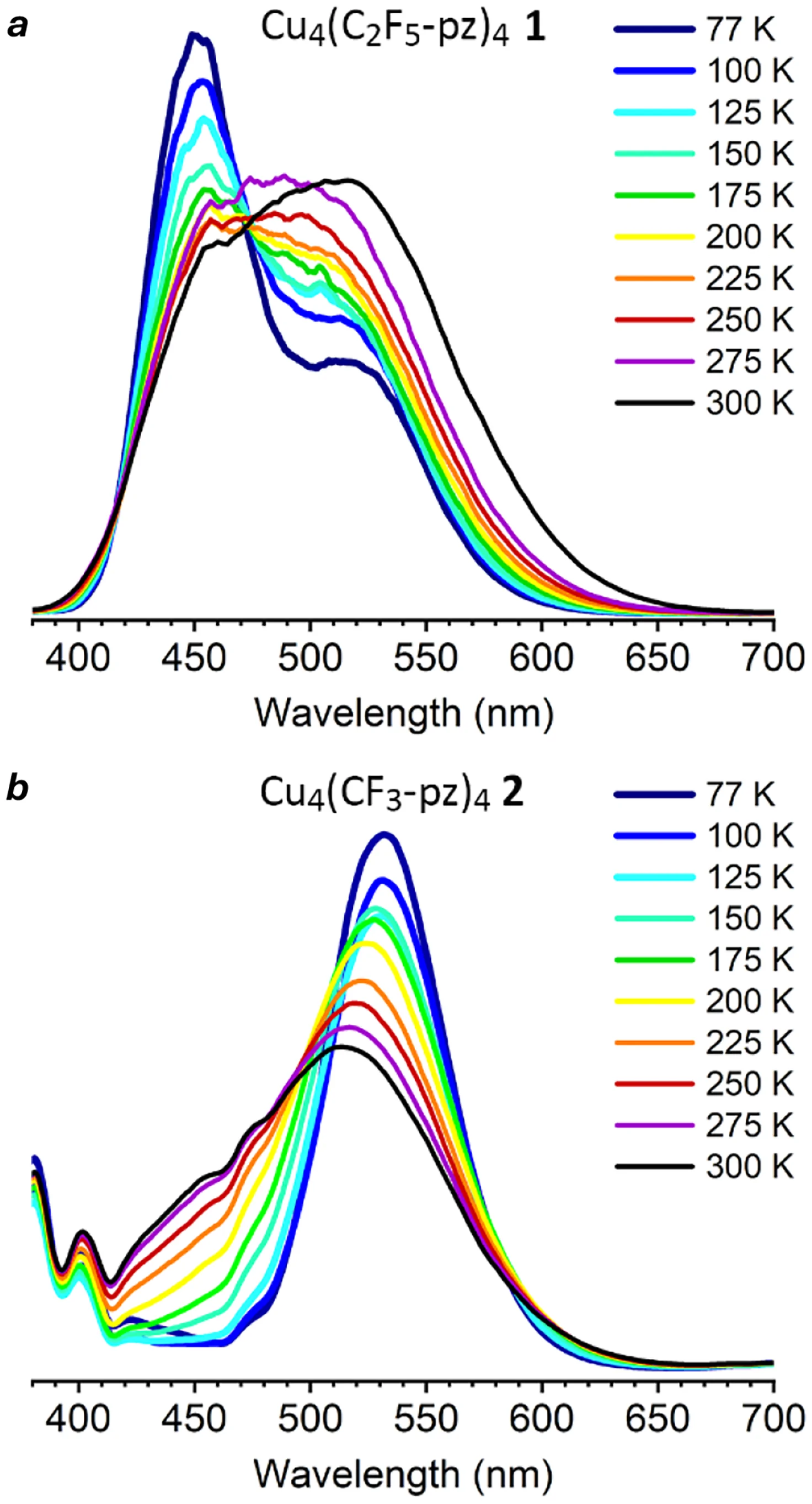
(a) VT-PL spectra of Cu_4_(C_2_F_5_-pz)_4_1 (thin film on quartz; *λ*_ex_ 270 nm) shows decrease in HE emission (450 nm) and increase in LE emission (520 nm) with temperature. (b) VT-PL spectra of Cu_4_(CF_3_-pz)_4_2 (*λ*_ex_ 270 nm) shows the opposite trend.^8^

The HE/LE ratio of 1 at 77 K is insensitive to *λ*_ex_, with the HE band being dominant for excitations between 250 and 300 nm (Fig. S3a, SI). PL intensities are at maximum for *λ*_ex_ at 280 nm, in accord with the excitation spectrum in Fig. 4b. The PL profiles are more affected by *λ*_ex_ at 300 K; although overall lineshapes are broad, additional features can be observed for *λ*_ex_ < 280 nm (Fig. S3b, SI), possibly due to locally excited states within the T_1_ manifold that become accessible at higher temperatures.

LE emission is presumably mediated through the T_1_ state, which is primarily CC in nature but has partial MLCT character (Fig. 7a and Table 1). The PL lifetime of the LE band is thermosensitive (*τ*_PL_ 49 → 38 µs for 77–300 K, Fig. 6a), attributable to vibronic coupling of the MLCT component. The HE band has a longer PL lifetime but even higher sensitivity to temperature (*τ*_PL_ 60 → 35 µs, Fig. 6b), indicating an excited state with greater MLCT character. An Arrhenius plot confirms the lower activation energy for PL decay of the HE band (0.038 eV) *versus* the LE band (0.052 eV) and suggests a thermally dependent crossover between the two emissive states (Fig. 6c).

**Fig. 6:**
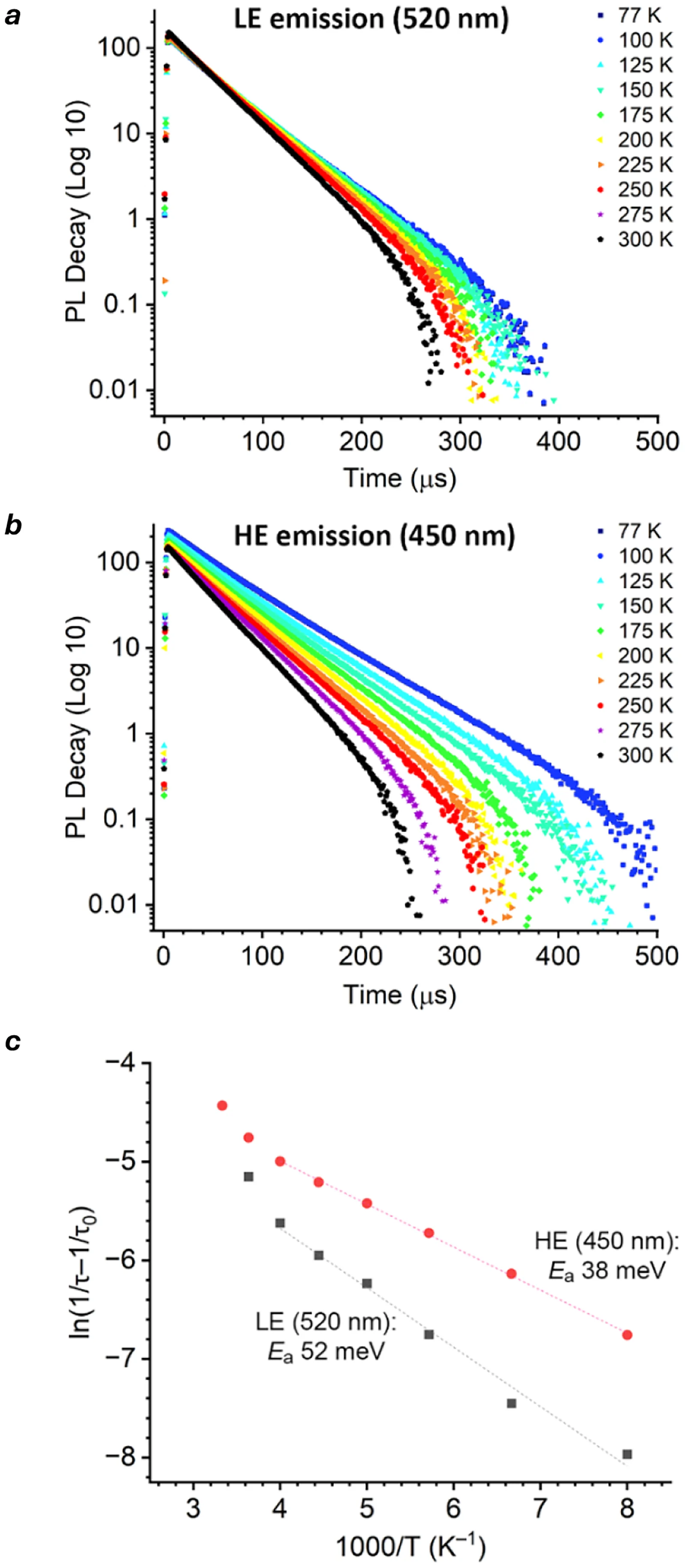
VT-PL lifetimes of Cu_4_(C_2_F_5_-pz)_4_1 (thin film on quartz; *λ*_ex_ 270 nm). (a) PL decay of LE band centred at 520 nm. (b) PL decay of HE emission band centred at 450 nm. (c) Arrhenius plot of ln(1/*τ*_PL_ − 1/*τ*_0_) showing lower activation energy (*E*_a_) for HE emission; *τ*_0_ values assigned to *τ*_PL_ at 77 K (48.6 μs for LE; 59.7 μs for HE).

**Fig. 7:**
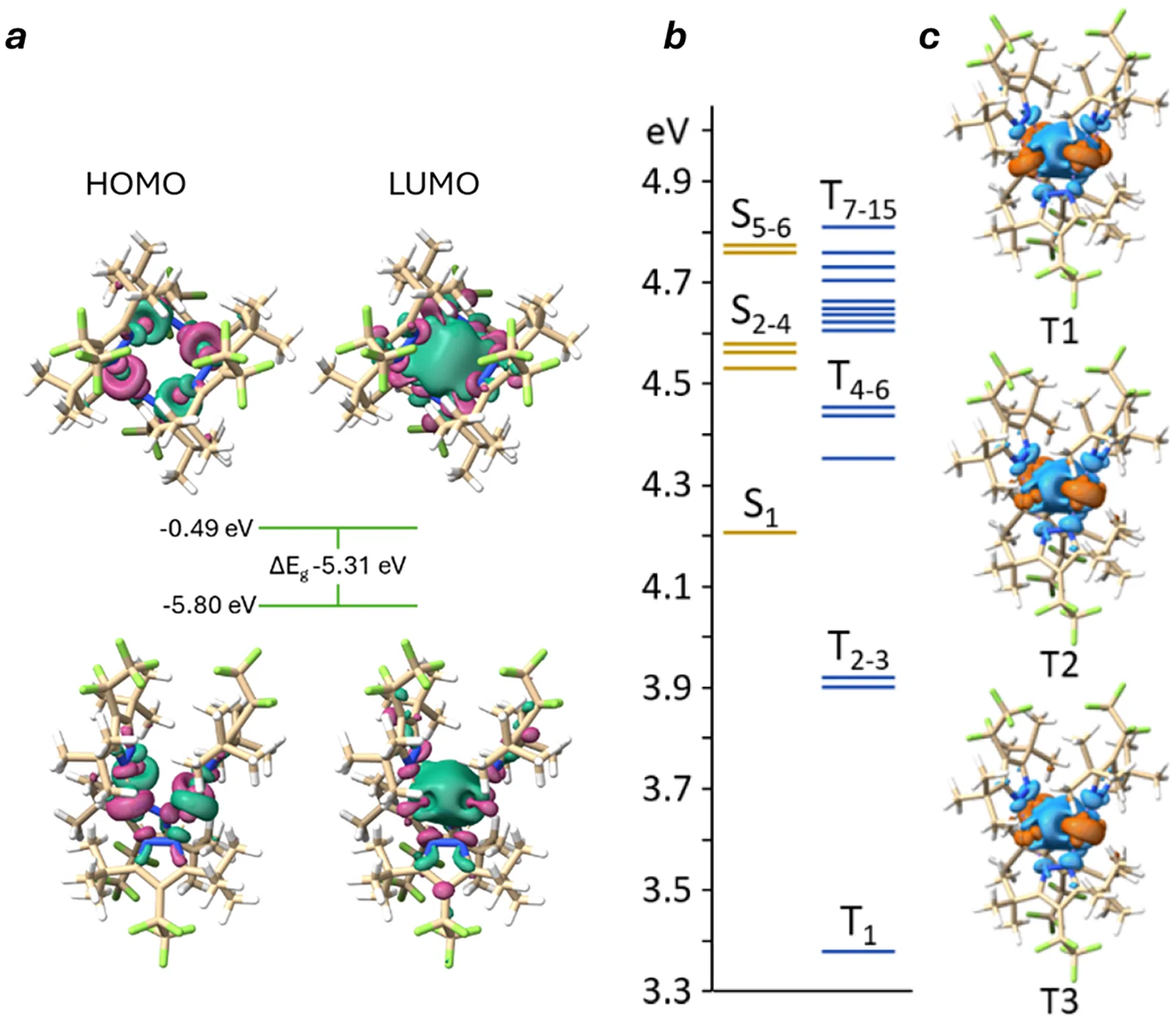
(a) Top and side views of CC states for 1 (LUMO and HOMO orbitals); (b) S_*m*_ and T_*n*_ vertical energies based on ground-state conformations; (c) electron density difference (EDD) maps for T_*n*_ → S_0_ (*n* = 1–3). Hole and electron domains are orange and blue, respectively.

**Table 1:** TD-DFT analysis of excited states in singlet and triplet manifolds of 1

Excited state(s)	Energy^a^ (eV)	*f* (10^−3^)	Major orbital contributions^b^ (%)	
			CC	MLCT
S_1_	4.22	1.50	40	34
S_2_	4.58	0.08	20	55
S_3_, S_4_	4.59	41.5	12	63
S_5_, S_6_	4.80	71.5	40	37
T_1_	3.39	—	46	29
T_2_, T_3_	3.91	—	42	35
T_4_	4.37	—	20	55
T_5_, T_6_	4.46	—	16	59

a Vertical energy from ground state.

b Quantified by the interfragment charge transfer method (Multiwfn); see SI for details.

Increases in PL intensity with temperature (often referred to as negative thermal quenching or NTQ) are uncommon and bear some discussion. Prior examples of NTQ in luminescent Cu(i) complexes are associated with low barriers between excited states, resulting in thermally activated delayed fluorescence (TADF)^30^ or phosphorescence.^31^ In the case of Cu_4_(C_2_F_5_-pz)_4_1, the LE band increases with temperature while the HE band decreases, with no net increase in overall PL intensity between 77 and 250 K (Fig. S2, SI). Thus, the LE and HE peak ratios most likely reflect changes in the relative populations within the triplet manifold prior to emission. While there are several possible ways to produce dual emission,^22,32^ the VT-PL study clearly supports competition between direct T_*n*_ emission (anti-Kasha) and T_1_ emission following internal conversion (IC).

### Computational studies

HE emissions from polynuclear Cu(i) clusters have been attributed to ^3^MLCT or ^3^LC states,^10,29^ halide–ligand charge transfer (XLCT) in Cu_4_X_4_ clusters, or ligand–ligand charge transfer (LLCT) in Cu(i) complexes designed for TADF.^33^ More recently, Dias *et al.* postulated the involvement of a high-lying ^3^CC state in HE emission from Cu_4_pz_4_ complexes in frozen solution.^14^ Here we examine this possibility in greater detail using TD-DFT and SOC matrix elements (SOCMEs) to elucidate the roles of S_*m*_ and T_*n*_ states in HE emission.

TD-DFT analysis of 1 was performed in the gas phase using the PBE0 hybrid functional and X2C-TZVPall basis set (see SI for details). Frontier orbital analysis of 1 indicates CC character in all excited-state pathways (S_0_ → S_*m*_ → T_*n*_ → S_0_), with variable contributions from pyrazolate ligands (Fig. 7a; Table 1). Electron density difference (EDD) maps confirm that transitions between T_*n*_ and S_0_ states are localized around the Cu_4_ core. However, the calculated energy gap between T_1_ and T_2_/T_3_ is over 0.5 eV (Fig. 7b, c; Table S3, SI) in accord with the experimental difference in HE and LE emissions of 0.37 eV. The large gap may be due to differences in excited-state bonding: in the T_1_ structure, all Cu atoms have filled d(*z*^2^) orbitals that maximize electronic interactions with pyrazolate ligands, whereas half of the Cu atoms in the T_2_ and T_3_ structures bond more weakly through d(*xz*) or d(*yz*) orbitals (Fig. S5; details in SI). We thus presume that T_*n*_ → S_0_ emission competes with IC,^20,31^ with both T_2_ and T_3_ contributing to the HE band.

The efficiency of intersystem crossing (ISC) between S_*m*_ and T_*n*_ states depends on the strength of spin–orbit coupling and the S–T energy difference, with a small gap enabling greater overlap between vibronic states.^34^ SOCME and Δ*E*_ST_ heat maps were produced for {S_*m*_,T_*n*_} pairs with vertical energies ≤4.6 eV (*λ*_ex_ 270 nm). S_3_ and S_4_ are the most favourable in the singlet manifold, with {S_3_,T_6_} and {S_4_,T_5_} having relatively large SOC and low Δ*E*_ST_ values of 39 cm^−1^ and 0.14 eV, respectively (Fig. 8). Several {S_1_,T_*n*_} pairs also have sizable SOCs, although their ISC rates may be reduced because of their larger Δ*E*_ST_. Overall, SOCME analysis supports ISC to high-lying T_*n*_ states, enabling emissions above T_1_.

**Fig. 8:**
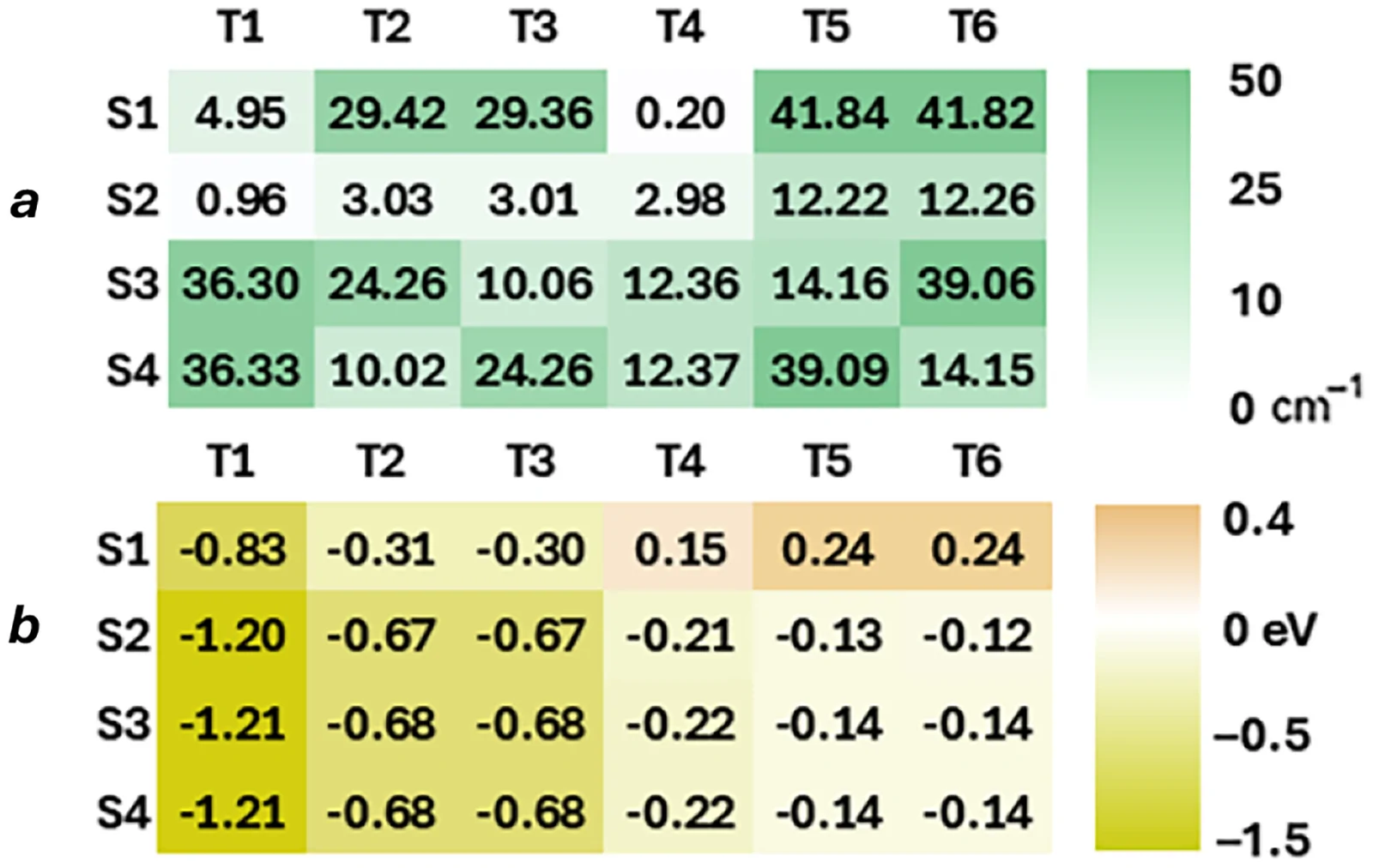
(a) Spin–orbit coupling energies (cm^−1^) between S_*m*_ and T_*n*_; (b) electronic energy differences (eV) between S_*m*_ and T_*n*_. Full SOCME and Δ*E*_ST_ heat maps in SI (Fig. S6).

Estimates of excited-state energies and electron densities are perturbed by transitions between ground- and excited-state conformations, which creates challenges for modelling emissions from the triplet manifold. To address this issue, triplet energies were recalculated using excited-state geometries optimized at T_1_, T_2_, and T_3_, which are presumed to be the most populated states prior to emission (Δ*E*_T1–T2_, Δ*E*_T1–T3_ = 0.08, 0.31 eV; Table S4, SI). For the T_1_- and T_3_-optimized geometry of 1, HE can be assigned to isoenergetic T_2_ and T_3_ states which lie above T_1_ (LE) by 0.5 eV. The situation is reversed for the T_2_-optimized structure: LE is represented by isoenergetic T_1_ and T_2_ states which lie below T_3_ (HE) by 0.4 eV. In all cases, the large energy gaps enable HE emission to compete with IC and subsequent LE emission. At low temperatures the IC barrier becomes more significant,^35,36^ further favouring HE emission (Fig. 5a).

It is interesting to consider the VT-PL behaviour of Cu_4_(CF_3_-pz)_4_2, which has the opposite temperature-dependent trend in HE/LE emission as 1 (Fig. 5b). Calculations of triplet energies from the excited-state geometries of 2 indicate similar degeneracies and energy gaps between T_1_ and T_2_/T_3_ or T_1_/T_2_ and T_3_, supporting bimodal emission (Table S5, SI). However, X-ray analysis of crystalline 2 indicates low barriers to conformational flux at low temperatures,^8^ which facilitates IC and the depopulation of high-lying triplet states as reflected by the dominant LE emission below 100 K. In turn, the population of higher T_*n*_ states and HE emission intensity increases with temperature in accord with the Boltzmann distribution.

Lastly, we emphasize that the blue emission from high-lying triplet states of 1 is distinct from that produced by other Cu_4_pz_4_ species *via* conformationally enforced rigidochromism.^7,9^ In those cases, the densely packed Cu_4_ core supports blue emission by minimizing excited-state contraction of the ^3^CC state. In the case of 1 (and 2), electronic energies within the triplet manifold are decreased by the inductive (electron-withdrawing) effects of C_*x*_F_*y*_ substituents: the S_0_ → T_1_ excitations for 1 and 2 (3.39 and 3.46 eV respectively) are 0.2 eV lower *versus* Cu_4_(CH_3_-pz)_4_ (3.60 eV).^7^ The electron-deficient pyrazolate ligands also increase the MLCT character of excited states, as has been observed for several Cu_3_(pz)_3_ species^37^ and Cu(phen)_2_ complexes.^38^ The inductive effect of the C_2_F_5_ group is presumed to be even stronger than that of CF_3_; although electrophilicity parameters for C_2_F_5_ are not yet available, photoelectron spectroscopy of perfluoroalkyl aromatics suggests a 55 meV difference in electron affinity between CF_3_ and C_2_F_5_ (>1 kcal mol^−1^).^39^

## Conclusions

Solid-state Cu_4_(C_2_F_5_-pz)_4_1 produces blue–green luminescence with high quantum yield, and crystallizes in prolate form with an all-*endo* conformation. Complex 1 displays dual emission at 450 and 520 nm that arises from its hybrid CC/MLCT character and large energy gap between triplet states. The electro-negative C_2_F_5_ groups impart a strong inductive effect that lowers electronic excited-state energies and increases access to high-lying states, enabling efficient intersystem crossing. Although steric crowding at the ligand periphery prevents the Cu_4_ core of 1 from adopting a close-packed geometry, deep-blue emission is still possible by using electronic tuning to enable anti-Kasha emission from high-lying triplet states.

## Author contributions

Shinaj Rajagopal: experimental results and analysis, writing – original draft. Christian Peterson: computational analysis, writing – SI. Matthias Zeller: X-ray crystallography and data analysis. Sergei Savikhin: experimental data analysis. Lyudmila Slipchenko: computational data analysis. Alexander Wei: supervision, data analysis, writing – review & editing.

## Conflicts of interest

There are no conflicts to declare.

## Supplementary Material

DT-055-D6DT01868H-s001

DT-055-D6DT01868H-s002

## Data Availability

Supplementary information (SI): all experimental procedures, characterization data, and the data underlying the figures. See DOI: https://doi.org/10.1039/d6dt01868h. Additional details will be made available upon request to the corresponding author. CCDC 2574785 (1) contains the supplementary crystallographic data for this paper.^40^
