# Supplementary material for: Electronic tuning of tetranuclear Cu(i)–pyrazolate macrocycles: anti-Kasha emission through high-lying triplet states of a C2F5-substituted complex
Source: Dalton Trans. 2026 Oct 13;55(42):15042–8. doi: 10.1039/d6dt01868h (PMC13673953; doi:10.1039/d6dt01868h)
Supplement: Supplementary file 2 [file DT-055-D6DT01868H-s001.pdf]

Supplementary Information (SI) for Dalton Transactions.
This journal is © The Royal Society of Chemistry

Electronic Supplementary Information

Electronic tuning of tetranuclear Cu(I)–pyrazolate macrocycles: Anti-Kasha emission through high-lying excited states of a C_2F_5-substituted complex

Shinaj K. Ragajopal,[1] Christian M. Peterson,[1] Matthias Zeller,[1] Sergei Savikhin,[2] Lyudmila V. Slipchenko,[1] and Alexander Wei[1,3,*]

[1] James and Margaret Tarpo Department of Chemistry, 560 Oval Drive, Purdue University, West Lafayette, IN 47907, USA

[2] Department of Physics and Astronomy, 525 Northwestern Avenue, Purdue University, West Lafayette, IN 47907, USA

[3] School of Materials Engineering, Purdue University, 701 W. Stadium Avenue, West Lafayette, IN 47907, USA

Table of Contents

1. General information, materials, and methods S2
2. Synthesis S3
3. Solid-state photoluminescence and quantum yield (Fig. S1–S3) S4
4. Single-crystal X-ray crystallographic data (Fig. S4; Tables S1, S2) S6
5. TD-DFT calculations (Fig. S5, S6; Tables S3–S5) S9
6. ^{1}H, ^{13}C, and ^{19}F NMR spectra (Fig. S7–S15) S12
7. References S16

1. General information and materials and methods

Chemical reagents and supplies obtained from commercial vendors were used as received unless otherwise noted. ^{1}H, ^{13}C and ^{19}F NMR spectra were collected at room temperature on a Varian INOVA 300 or Bruker NEO500 spectrometer. NMR samples were prepared in chloroform-*d* with chemical shifts (δ) reported in ppm relative to residual non-deuterated solvent resonances as internal standards. Full details on the synthesis and characterization of $Cu_4(C_2F_5pz)_4$ complex **1** and 3,5-di-^{t}Bu-4-pentafluoroethylpyrazole **3** are described in the respective sections below.

Single-crystal samples for x-ray diffraction were grown in CH_2Cl_2–toluene mixtures with slow evaporation at rt. Thin-film samples were prepared on quartz substrates cleaned ultrasonically with toluene, acetone and 2-propanol, followed by plasma treatment for 10 min using a Harrick plasma cleaner PDC-XG3. Cu_4pz_4 complexes in dichloromethane (5 mg/mL) were filtered through a 0.2-μm PTFE membrane (Whatman), cast onto quartz substrates using a SPS Polos SPIN 150i spin coater (5000 rpm, 30 s), then dried at rt for 30 min under vacuum (Fisher Scientific Isotemp® Vacuum Oven, Model 281A).

Absorption spectra were acquired on a Varian Cary50 Bio UV-visible spectrophotometer using a 1-cm quartz cuvette. Photoluminescence (PL) spectra and absolute quantum yields (Φ_{PL}) were measured using an Edinburgh Instruments FLS 980 spectrometer with an integrating sphere accessory. PL lifetimes (τ) and variable-temperature analyses were measured using a Varian Cary Eclipse spectrophotometer using a quartz tube (5 mm outer diameter, 4 mm inner diameter, 77 mm tube length, Wilmad-LabGlass) or a quartz substrate using an excitation wavelength of 270 nm; τ values of solid-state samples were obtained from mono-exponential fits to the data at room temperature (297 K).

Quantum chemical calculations were performed using a hybrid density functional theory (DFT) functional (ORCA 6.1.1).[S1] Geometries of Cu_4pz_4 complexes were optimized in the gas phase using PBE0/X2C-TZVPall for the method/basis set with D4 dispersion included. The X2C relativistic Hamiltonian was chosen to handle relativistic effects, and the X2C/J auxiliary basis set was called to enable RIJCOSX approximation. Fine integration with DEFGRID3 and stringent convergence criteria with TightSCF were utilized throughout. Time-dependent DFT (TD-DFT) was performed with the same functionals for consistency, and the spin–orbit coupling (SOC) operator was enabled to obtain SOCME values. Electron density difference (EDD) surfaces were generated with the associated utility in orca_plot and visualized using ChimeraX.[S2] Charge transfer analysis was performed using Multiwfn[S3] and the wavefunction information provided by ORCA.

2. Synthesis

1-Benzyl-3,5-di-*tert*-butyl-4-pentafluoroethyl-1*H*-pyrazole (1-Bn-3,5-di-^{t}Bu$_2$-4-C_2F_5-pz)

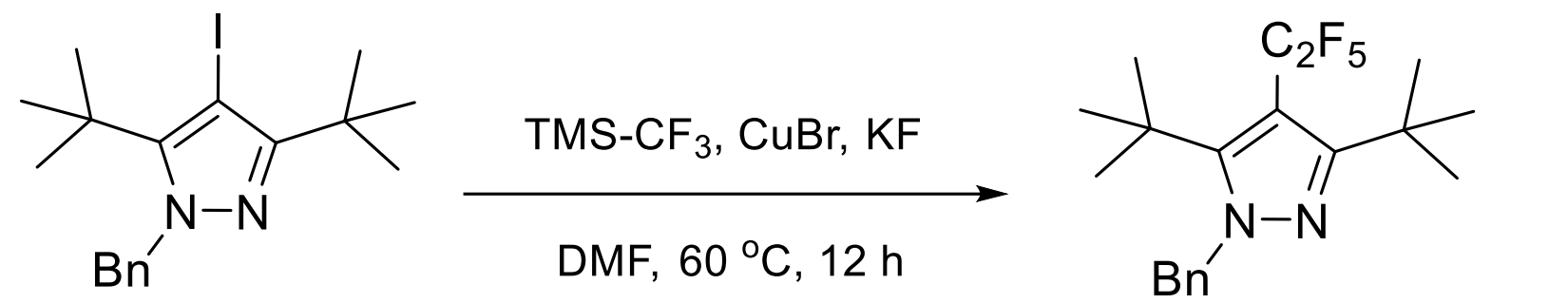

1-Bn-3,5-^{t}Bu$_2$-4-I-pz   1-Bn-3,5-^{t}Bu$_2$-4-(C_2F_5)-pz

1-Benzyl-3,5-di-*tert*-butyl-4-iodo-1*H*-pyrazole was prepared according to our previous procedure.[S4] Pentafluoroethylation was performed using the method described by Boutureira and co-workers.[S5] CuC_2F_5 was generated by treating predried CuBr (1.0 g, 7 mmol) and KF (324 mg, 5.6 mmol) in anhydrous DMF (24.5 mL) with stoichiometric $TMSCF_3$ (0.83 g, 5.6 mmol), followed by slow heating to 55 °C and stirring for 1.5 days under argon. In a separate flask, 4-iodopyrazole (1.0 g, 2.52 mmol) was added to anhydrous DMSO (0.5 mL) and heated to 60 °C under argon, then treated with freshly prepared CuC_2F_5 solution and stirred for 12 h. The reaction mixture was treated with saturated aq $NaHCO_3$ (100 mL) at rt, extracted with EtOAc and washed with brine, then dried with Na_2SO_4 and concentrated by rotary evaporation. Silica gel chromatography using 30% CH_2Cl_2 in hexanes yielded **1-Bn-3,5-di-^{t}Bu-4-C_2F_5-pz** as a white solid (0.80 g, 82%). ^{1}H NMR: δ 7.30 (t, *J* 7.3 Hz, 2H), 7.24 (t, *J* 7.3 Hz, 1H), 6.93 (d, *J* 7.3 Hz, 2H), 5.59 (s, 2H), 1.38 (s, 9H), 1.35 (s, 9H). ^{19}F NMR (101 MHz, $CDCl_3$): –78.95 (s). ^{13}C NMR (126 MHz, $CDCl_3$): δ 158.21, 152.13, 138.11, 128.55, 127.20, 126.09, 119.67 (tq, *J* 40, 288 Hz), 114.43 (qt, *J* 40, 226 Hz), 104.04 (t, *J* 30.2 Hz), 57.02, 35.19, 34.01, 31.67, 30.90.

3,5-Di-*tert*-butyl-4-(pentafluoroethyl)-1*H*-pyrazole (3,5-^{t}Bu$_2$-4-C_2F_5-pz) (3)

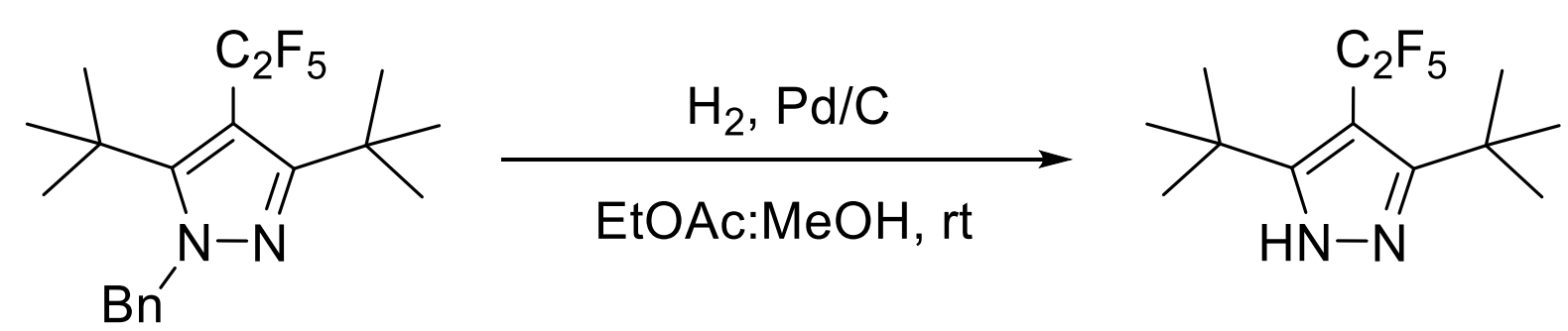

1-Bn-3,5-^{t}Bu$_2$-4-C_2F_5-pz   3,5-^{t}Bu$_2$-4-C_2F_5-pz (**3**)

1-Bn-3,5-di-^{t}Bu-4-C_2F_5-pz (0.80 g, 2.06 mmol) was dissolved in 1:1 methanol:ethyl acetate (16 mL) and treated with 5% palladium on charcoal (500 mg, 0.21 mmol). The mixture was charged several times with H_2 then stirred for 1 hour at rt and 1 atm. The reaction mixture was filtered over Celite, concentrated and passed through a silica gel plug (20% EtOAc:Hexane) to afford pyrazole **3** as a white solid (0.40 g, 65%). ^{1}H NMR (500 MHz, $CDCl_3$) δ 1.36 (18H, s). ^{19}F NMR (101 MHz, $CDCl_3$): δ –79.44 (s), –97.16 (s). ^{13}C NMR (126 MHz, $CDCl_3$) δ 156.80, 119.33 (tq, *J* 41.6, 288.5 Hz), 114.51 (qt, *J* 41.6, 253.3 Hz), 102.98 (t, *J* 30.2 Hz), 34.25, 30.61. HR-ESIMS: calcd. *m/z* for $C_{13}H_{20}F_5N_2$ $[M+H]^+$ = 299.1542; observed $[M+H_3O]^+$ 299.1540.

Cu_4(3,5-^{t}Bu_2-4-C_2F_5-pz)$_4$ complex (1)

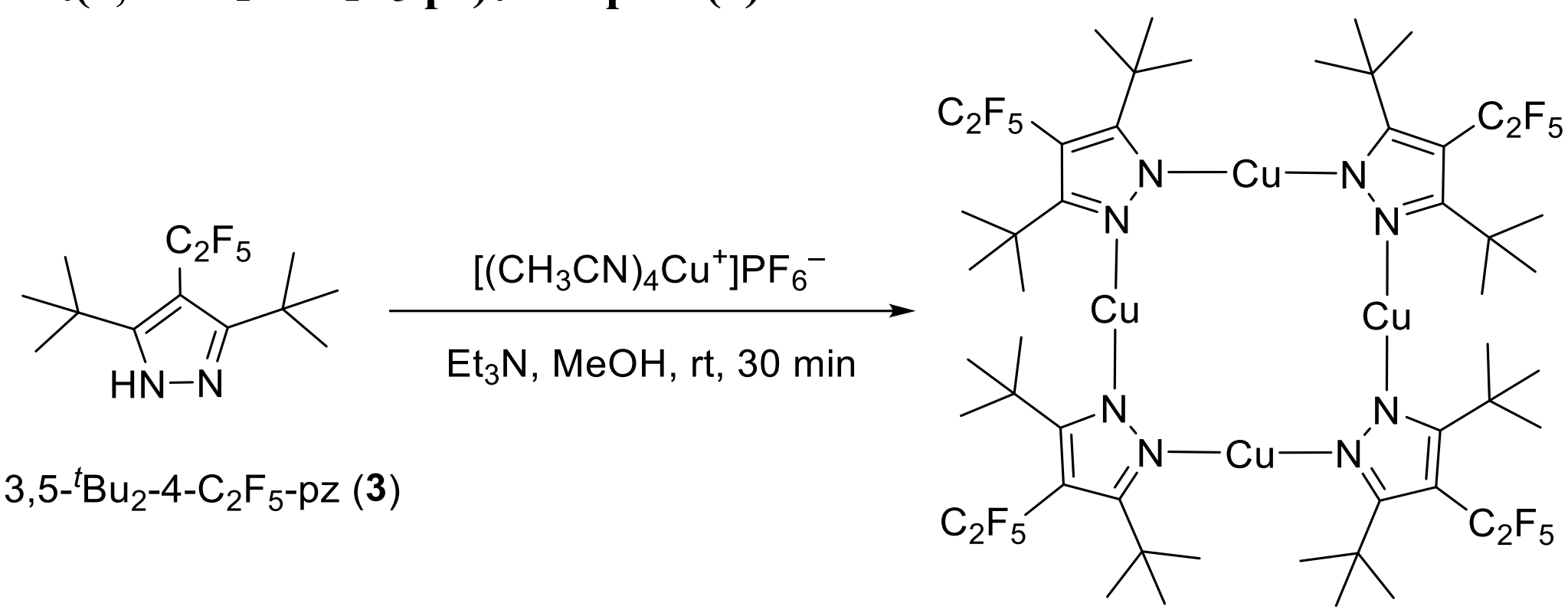

3,5-^{t}Bu_2-4-C_2F_5-pz (**3**)

$Cu_4(C_2F_5\text{-pz})_4$ (**1**)

Methanol and triethylamine (Et_3N) were freshly distilled and degassed prior to use. C_2F_5-pyrazole **3** (100 mg, 0.335 mmol) was added to a flame-dried Schlenk flask with stir bar under argon and dissolved in methanol (4.5 mL). The solution was degassed with argon then treated with $[(CH_3CN)_4Cu^+]PF_6^-$ (127 mg, 0.335 mmol) and stirred for 5 minutes, followed by the dropwise addition of degassed Et_3N (186 µL, 1.34 mmol). The reaction mixture was stirred under argon for another 30 minutes to produce a precipitate, which was filtered, washed with methanol and hexanes, and dried under vacuum to yield $Cu_4(C_2F_5\text{-pz})_4$ complex **1** as a colorless solid (105 mg, 87%). ^{1}H NMR (500 MHz, $CDCl_3$): δ 1.43 (s). ^{19}F NMR (101 MHz, $CDCl_3$): δ –76.38 (s), –91.07 (s). ^{13}C NMR (126 MHz, $CDCl_3$) δ 161.40, 119.66 (tq, *J* 42.8, 289.8 Hz), 115.59 (qt, *J* 39.0, 254.5 Hz), 103.69 (t, *J* 30.2 Hz), 34.33, 32.01. Combustion analysis calc. for $C_{52}H_{72}Cu_4F_{20}N_8$: C, 43.85; H, 5.09; N, 7.87; found: C, 43.36; H, 5.02; N: 7.72.

3. Solid-state photoluminescence (PL) and quantum yield

The PL quantum yield (Φ_{PL}) is the ratio of the number of photons emitted (N_{em}) to the number of photons absorbed (N_{abs}). A thin-film sample cast on a 7 × 10 mm quartz substrate was fixed onto a 10-mm diameter tray made from PTFE with quartz coverlets that are part of the FLS 980's integrating sphere assembly; a same size quartz substrate was used as a reference. The bandwidths for excitation wavelength ($\Delta\lambda_{ex}$) and emission wavelength ($\Delta\lambda_{em}$) are 10 nm and 0.1 nm, respectively. Spectral measurements were collected with direct excitation of the sample (E_A and S_A) and with indirect excitation (E_B and S_B) by using scattered photons in the spherical chamber (**Figure S1**), and corrected with measurements of the reference (E_{ref} and S_{ref}). Spectral scans of excitation scatter (S_{ref}, S_A and S_B) and emission (E_{ref}, E_A and E_B) were used to calculate Φ_{PL} by the direct and indirect excitation method, according to Equation S1:

$$\Phi_{PL} = \frac{N^{em}}{N^{abs}} = \frac{S_A(E_B - E_{ref.}) - S_B(E_A - E_{ref.})}{(S_A - S_B)S_{ref.}}$$

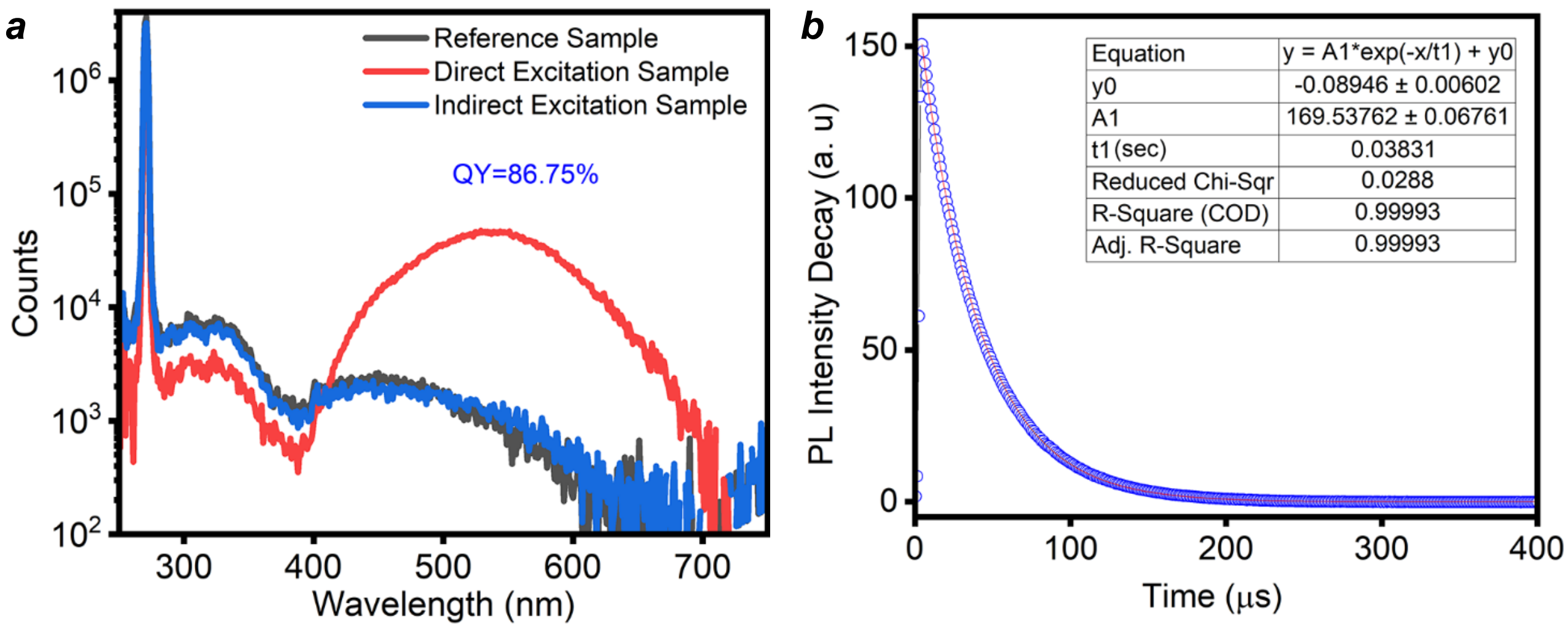

Figure S1. (a) PL quantum yield analysis of $Cu_4(C_2F_5\text{-pz})_4$ **1** (thin film on quartz) at 300 K (λ_{ex} 270 nm), based on spectral scans of excitation (scatter) and emission regions; Φ_{PL} = 87%. Purple trace = powder samples; yellow/blue traces = controls (blank/off-peak excitations). (b) Transient decay curve of **1** for PL lifetime analysis at 300 K; τ = 38.3 μs.

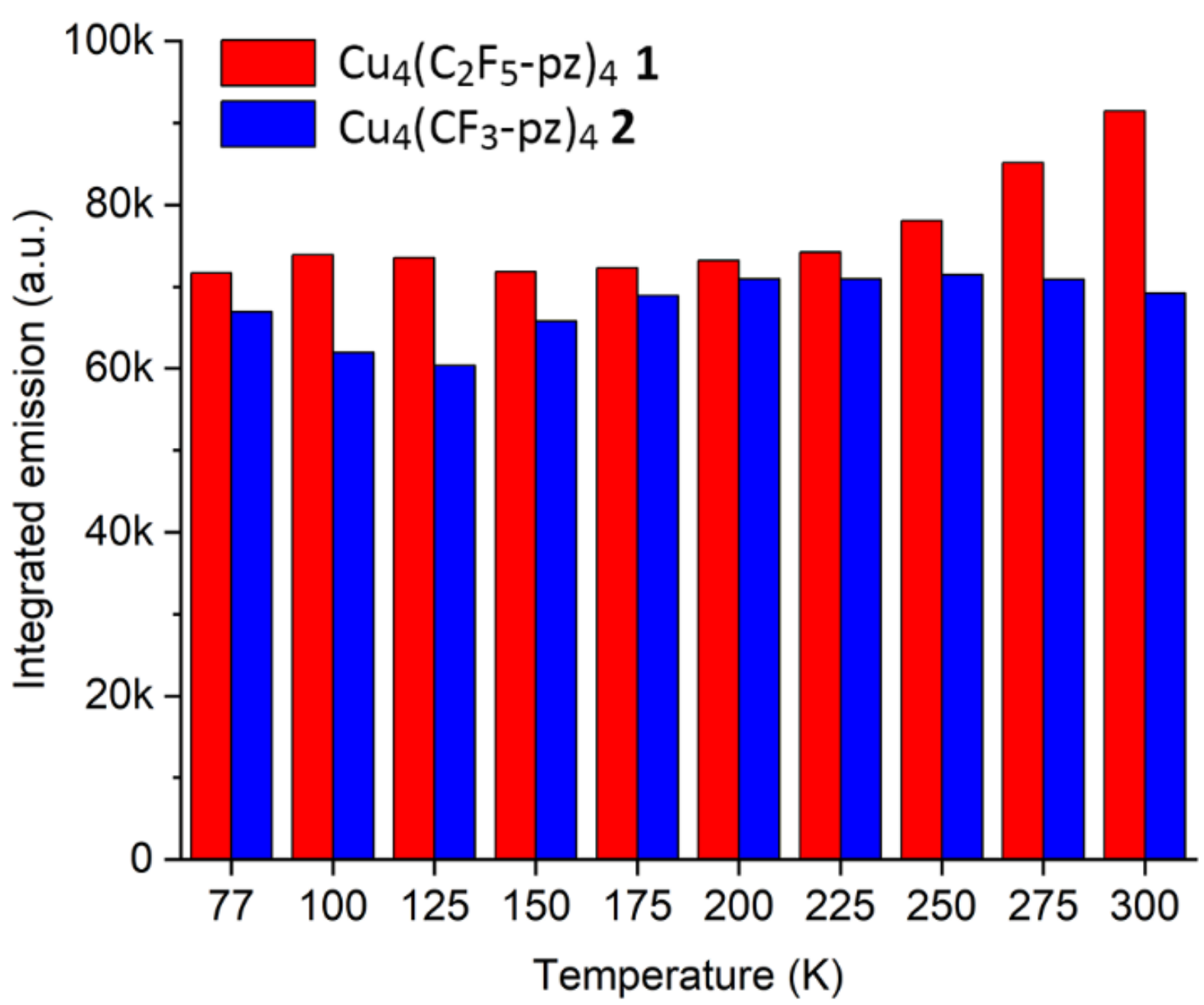

Figure S2. Integrated emission intensities of **1** (red) and **2** (blue) between 77 and 300 K, based on the VT-PL data in Figure 5a and 5b, respectively. PL spectra of **1** integrated from 400 to 700 nm; PL spectra of **2** integrated from 390 to 650 nm.

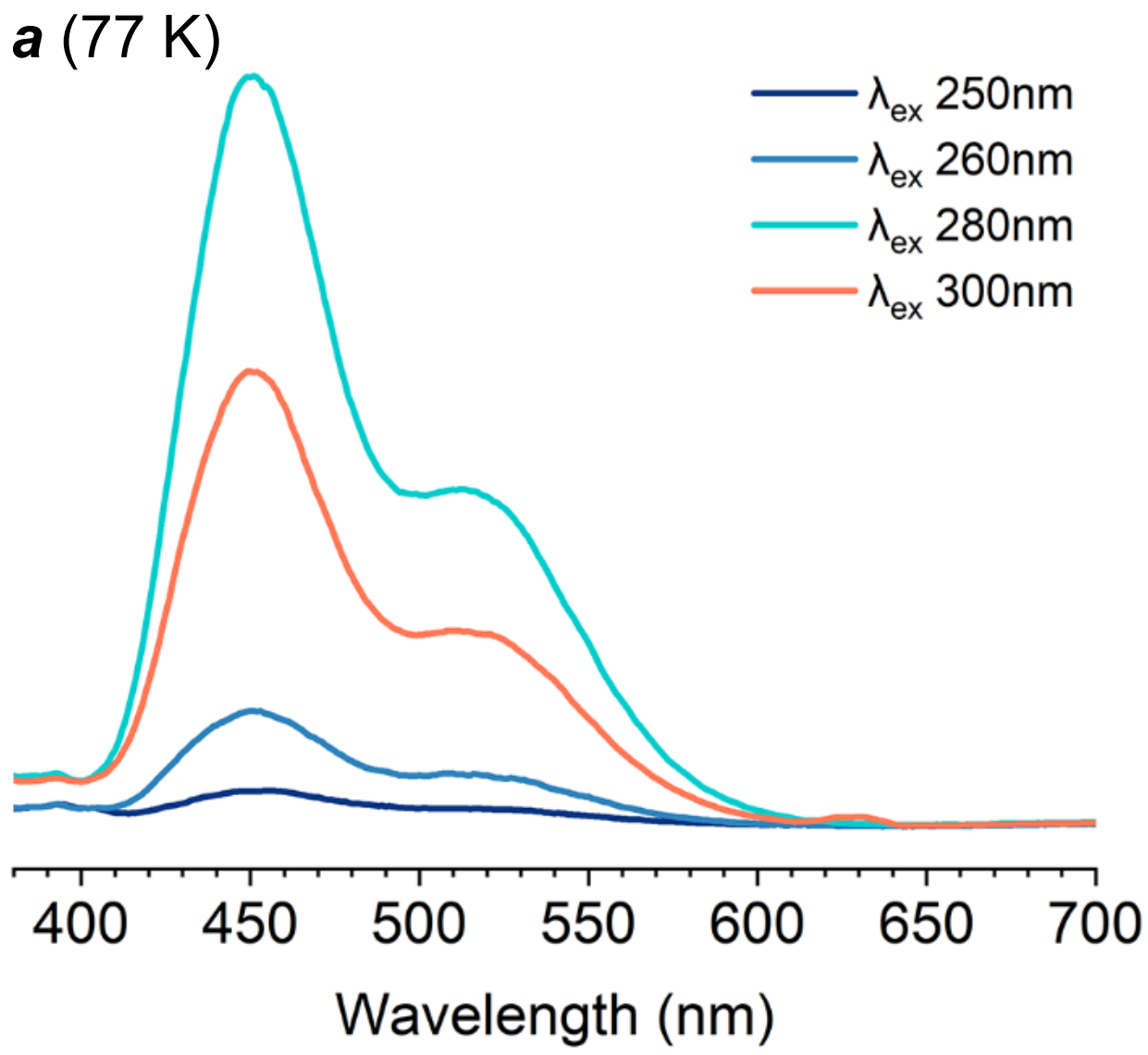

Figure S3. PL spectra of **1** (thin film) excited at select wavelengths between 250 and 300 nm. (a) acquired at 77 K; (b) acquired at 300 K.

4. Single-crystal X-ray crystallographic data

Single-crystal X-ray diffraction studies were carried out at the Purdue X-Ray Crystallography Facility using a Bruker AXS D8 Quest CMOS diffractometer. Single crystals of $Cu_4(C_2F_5\text{-pz})_4$ complex **1** were grown by slow solvent evaporation in dichloromethane/toluene mixture at room temperature. Crystals were coated with Fomblin oil (Solvay) and quickly transferred to the goniometer head of a Bruker Quest diffractometer with a fixed chi angle, a sealed tube fine focus X-ray tube, single crystal curved graphite incident beam monochromator, a Photon II area detector and an Oxford Cryosystems low temperature device. Examination and data collection were performed with Mo *K*α radiation (λ = 0.71073 Å) at 150 K. Complete crystallographic data in CIF format has been deposited with the Cambridge Crystallographic Data Centre (CCDC 2574785). These data can be obtained free of charge from the Cambridge Crystallographic Data Centre via www.ccdc.cam.ac.uk/data_request/cif.

For all crystal data collected, reflections were indexed and processed with files scaled and corrected for absorption using APEX3.[S6] Space groups were assigned and structures were solved by direct methods using XPREP within the SHELXTL suite of programs and refined by full-matrix least squares against F^2 with all reflections using SHELXL-2018 with graphical interface ShelXle.[S7,S8,S9] H atoms were positioned geometrically and constrained to ride on their parent atoms, with carbon–hydrogen bond distances of 0.95 Å for aromatic C–H, and 0.99 and 0.98 Å for aliphatic CH_2 and CH_3 moieties, respectively. $U_{iso}(H)$ values were set to a multiple of $U_{eq}(C)$ with 1.2 for CH_2 and 1.5 for CH_3 units, respectively.

Alternative geometries are observed for one pyrazolate ligand with no disorder noted in the Cu atoms. As such, the nitrogens of the disordered pyrazole were set to have identical coordinates. The primary conformer was found to be the all-*endo* conformer. The disordered pyrazole has its C_2F_5 group both pointing *endo* and *exo* with respect to the Cu cluster, generating a minor tri-*endo*

conformer. The disordered groups are restrained to have similar geometries; U_{ij} components of ADPs for disordered atoms closer than 2.0 Å are also restrained to be similar. Subject to these conditions the occupancy ratios are refined to 0.606(4) to 0.394(4) for N1 to F5B in Residue 3. The final renderings are presented in Figure S4.

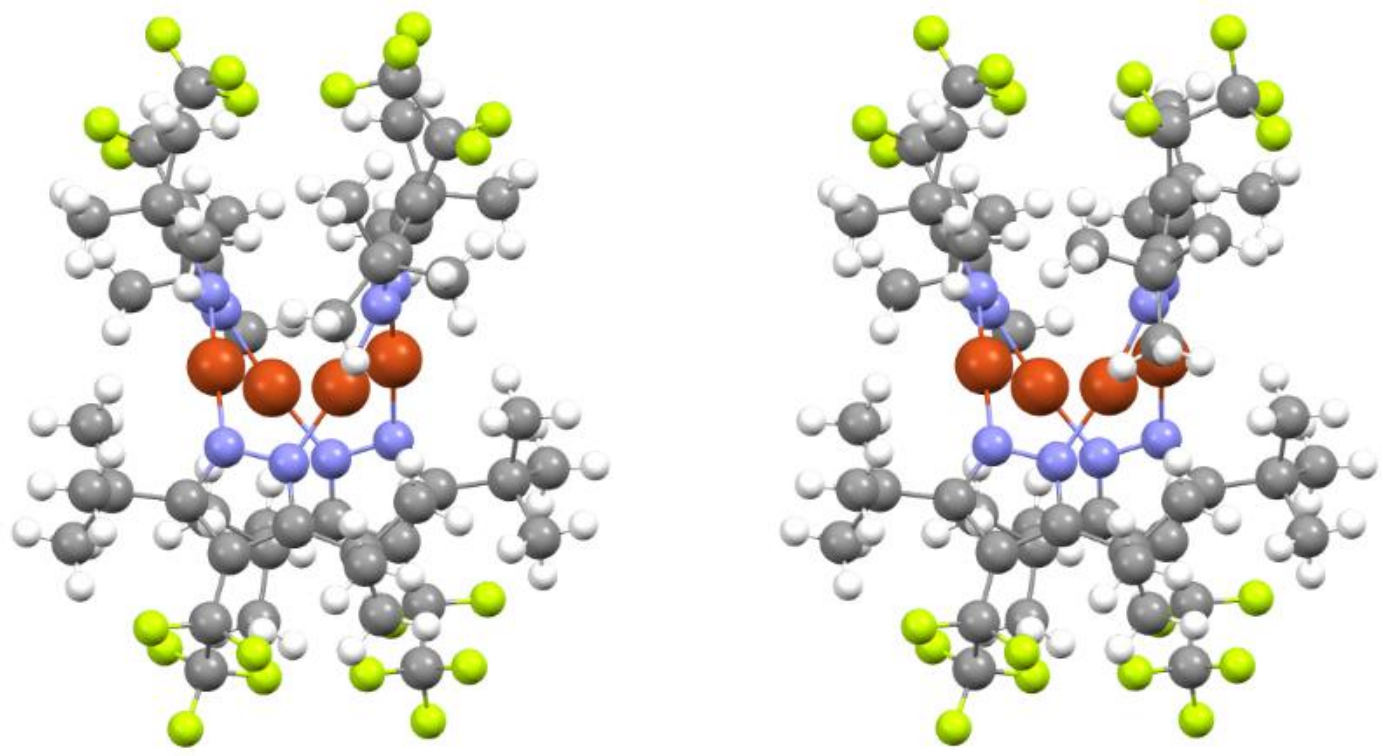

Figure S4. Major and minor conformers in $Cu_4(C_2F_5\text{-pz})_4$ polymorph **I** (150 K), with C_2F_5 substituents in all-*endo* and tri-*endo* orientations, respectively.

Table S1. Various geometric parameters for $Cu_4(C_2F_5\text{-pz})_4$ complex **1** at 150 K.

Adjacent Cu···Cu distance (Å) [a]	2.837(1)<br>2.811(1)<br>2.824(1)<br>2.782(1)
Diagonal Cu···Cu distance (Å):	3.980 (d_L)<br>3.847 (d_S)
Cu_4 quadrangle aspect ratio (d_L/d_S)	1.035
Cu···Cu···Cu bond angles (°)[b]	86.3<br>90.0
Cu_4 quadrilateral bend angle (°)[b,c]	28.4
Twist angles between pyrazolates (°)[d]	31.6<br>34.6

[a] Equivalent standard deviations (ESDs) for bond distances between adjacent Cu atoms are provided in parentheses. [b] Mean of two measurements. [c] Angle at intersection of planes defined by Cu_1–Cu_2–Cu_3 and Cu_1–Cu_4–Cu_3, or by Cu_2–Cu_3–Cu_4 and Cu_2–Cu_1–Cu_4. [d] Torsional angle between transannular pyrazolate N–N bonds (N–N···N–N).

Table S2. Crystal data and structure refinement for $Cu_4(C_2F_5\text{-pz})_4$ **1** at 150 K

**Crystal data**	
Chemical formula	C52 H72 Cu4 F20 N8
*M*r	1443.38
Crystal system, space group	Orthoclinic, *Pccn*
Temperature (K)	150
a, b, c (Å)	19.582(5), 42.415(11), 14.881(2)
α, β, γ (°)	90, 90, 90
V (Å^3)	12360(5)
Z	8
Radiation type	Mo *K*α
μ (mm^{-1})	1.567
Crystal size (mm^3)	0.180 × 0.110 × 0.060
**Data collection**	
Diffractometer	Bruker AXS D8 Quest with PhotonII charge-integrating pixel array (CPAD)
Absorption correction	Multi-scan SADABS 2016/2[S9]
T_{min}, T_{max}	0.618, 0.754
No. of measured, independent and observed [$I > 2\sigma(I)$] reflections	150991, 13478, 12239
R_{int}	0.0771
$(\sin \theta/\lambda)_{max}$ ranges (Å^{-1})	80.660
**Refinement**	
R [$F^2 > 2\sigma(F^2)$], $wR(F^2)$, S	0.0593, 0.1525, 1.140
No. of reflections	13478
No. of parameters	932
H-atom treatment	H-atom parameters constrained
$\Delta\rho_{max}$, $\Delta\rho_{min}$ (e Å^{-3})	0.757, -0.488

5. TD-DFT calculations

Coordinates from the crystal structure of $Cu_4(C_2F_5\text{-pz})_4$ complex **1** (150 K) and $Cu_4(CF_3\text{-pz})_4$ complex **2** (150 K) were used in the initial geometries for calculations, which were fully optimized at the triple-zeta level for H, C, N, F, and Cu atoms. For higher excited states, pre-optimization was performed at the double-zeta level to expedite arrival to an initial geometry, followed by triple-zeta optimization. Excited-state geometries were also optimized for vertical emission predictions using TD-DFT, to maintain consistency with the TD-DFT workflow. The PBE0 functional was chosen over a range-separated hybrid to emphasize local excitation, and the X2C method was favored over the DKH class of Hamiltonians because of its exact decoupling in a two-component relativistic framework.[S10] In addition, the X2C-TZVPall basis set provided superior performance for electron density calculations at relatively low computational cost.[S11]

Vertical excitation energies were determined from single-point calculations at the S_0 geometry, whereas vertical emission energies were obtained at the T_1-optimized geometry. SOC values were computed at the S_0 geometry under Franck–Condon assumptions, and the resulting S_m–T_n coupling vectors were reduced to their magnitudes as reported in the SOCME matrix (Figure S6). High- and low-energy triplet manifolds were distinguished based on their electronic couplings, as inferred from the SOC-corrected state composition. Charge-transfer characteristics were quantified from TDDFT-generated wavefunctions using Multiwfn for interfragment charge-transfer (IFCT) analysis based on the Hirshfeld partition method.[S3] Fragments assigned as Cu, pyrazolyl (pz), and C_2F_5, with ^{3}CC character defined by the electron redistribution within the Cu_4 core and ^{3}MLCT character defined as $H_{Cu}\cdot[E_{pz} + E_{C2F5}]$ with H and E representing hole and electron in decimal form.

Excited-state structure and bonding were evaluated by examining the highest occupied natural transition orbitals (HONTO) of the optimized T_1, T_2, and T_3 states (**Figure S5**). All Cu orbitals in the HONTO of T_1 have $d(z^2)$ configuration, which provides maximum orbital overlap with the pyrazolate nitrogen atoms and is typical of linear η^2-coordination. In the HONTO of T_2 and T_3, all Cu atoms have predominantly $d(xz)$ or d(yz) orbitals which bond less strongly to their adjacent nitrogens. The neighbouring pyrazolate ligands also exhibit well-defined N=N π-bonds, reflecting the lower contribution of p-orbital density to the weakened Cu–N bonds. We thus attribute the difference in energies between T_1 and T_2/T_3 to the overall stability of the excited-state structures.

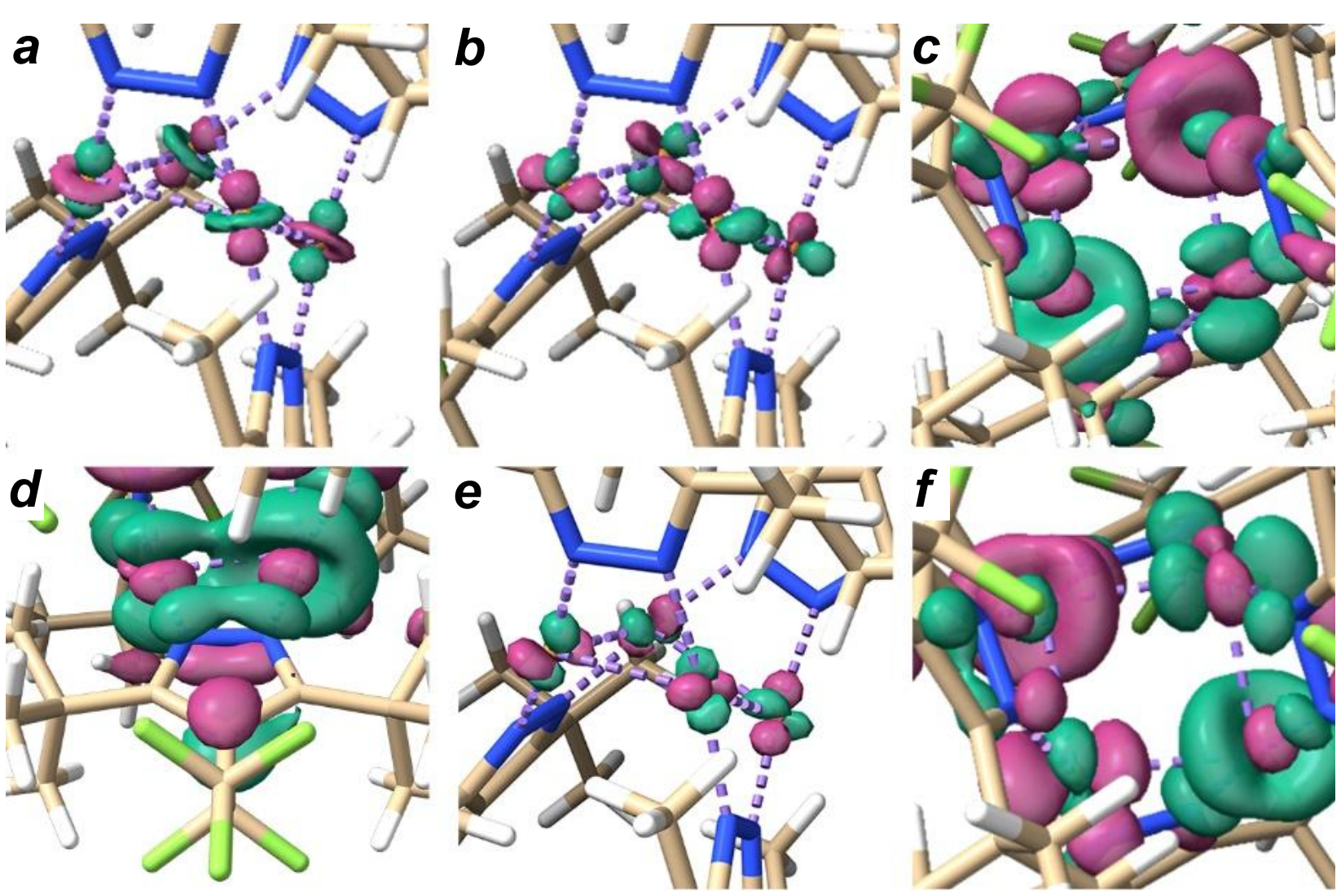

Figure S5. (a) HONTO in T_1 state of **1** (isovalue ±0.10 au), showing Cu atoms with $d(z^2)$ orbitals; (b) HONTO in T_2 (isovalue ±0.10 au) showing Cu atoms with $d(yz)$ orbitals; (c) HONTO in T_2 (isovalue ±0.03 au) showing two Cu atoms with significant $d(z^2)$ character; (d) HONTO in T_2 (isovalue ±0.03 au) showing π-bond between pyrazolate N atoms; (e,f) HONTO in T_3, showing Cu atoms with $d(xz)$ orbitals (isovalue ± 0.10 au) and two Cu atoms with significant $d(z^2)$ character (isovalue ± 0.03 au).

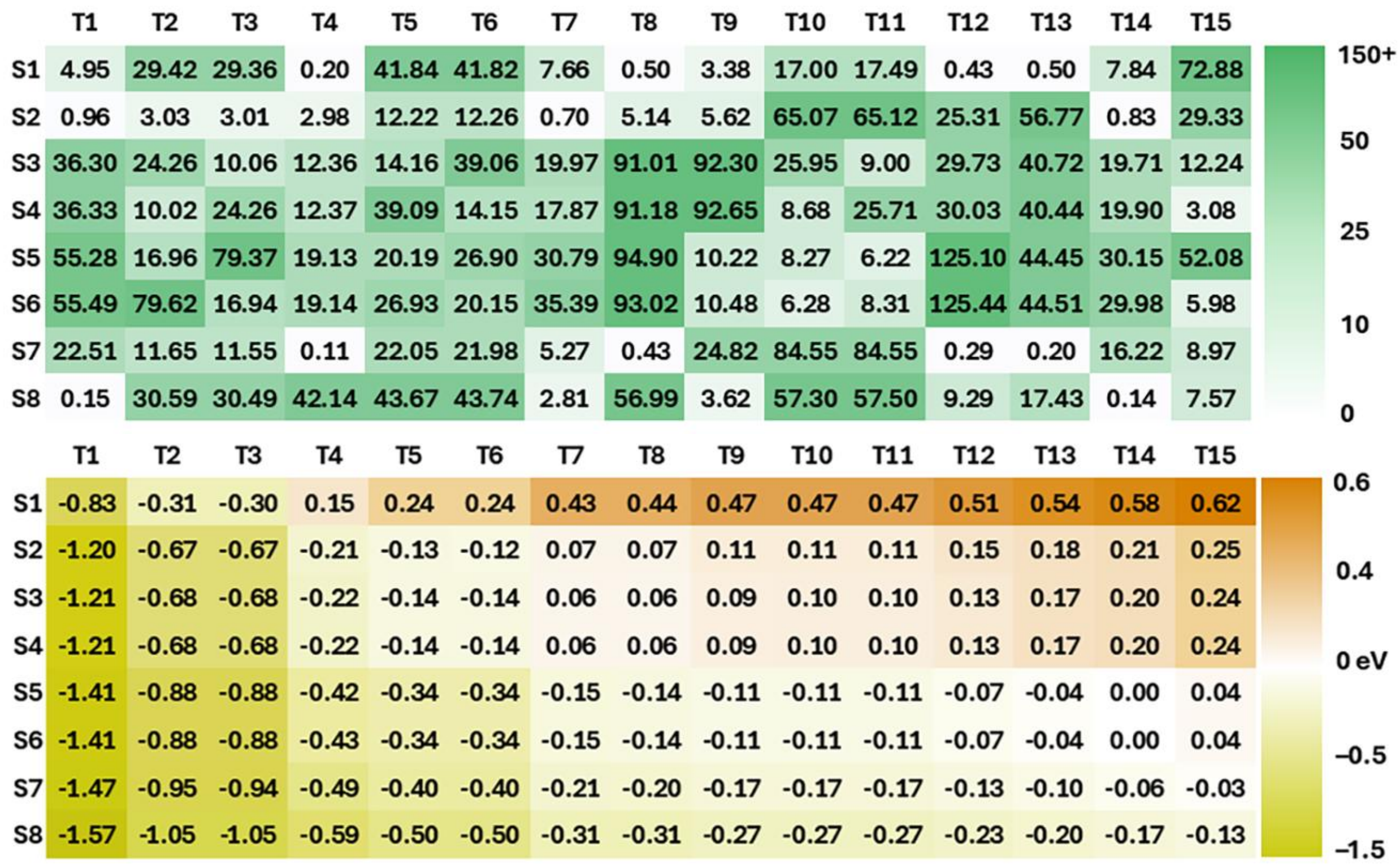

Figure S6. (a) Spin-orbit coupling matrix element (SOCME) energies (cm^{-1}) between S_m and T_n of $Cu_4(C_2F_5\text{-pz})_4$ **1**; (b) singlet–triplet energy differences (ΔE_{ST}, in eV) between S_m and T_n.

Table S3. Select $S_0 \rightarrow S_m$ and $S_0 \rightarrow T_n$ transitions, energies (E_{calc}), and major orbital contributions for $Cu_4(C_2F_5\text{-pz})_4$ **1** from TD-DFT calculations.

State	E_{calc} (eV)	Orbital contributions
S_1	4.216	HOMO → LUMO (92%)
S_2	4.580	HOMO → LUMO+3 (88%)<br>HOMO-1 → LUMO+2 (3%)<br>HOMO-2 → LUMO+1 (3%)
S_3	4.592	HOMO → LUMO+1 (86%)<br>HOMO → LUMO+2 (5%)
T_1	3.385	HOMO → LUMO (80%)<br>HOMO → LUMO+6 (5%)<br>HOMO → LUMO+4 (4%)
T_2	3.911	HOMO-1 → LUMO (58%)<br>HOMO → LUMO+1 (6%)<br>HOMO → LUMO+2 (6%)
T_3	3.912	HOMO-2 → LUMO (58%)<br>HOMO → LUMO+2 (6%)<br>HOMO → LUMO+1 (6%)

Table S4. Vertical emissions from excited-state geometries of **1**, optimized using TD-DFT

State	HE Band	LE Band	E_{calc}, in eV (λ)	$E_{T1} - E_{Tn}$ (eV)
T_1 (opt)	T_2 / T_3	T_1	HE: 3.04 eV (408 nm)<br>LE: 2.52 eV (492 nm)	--
T_2 (opt)	T_3	T_1 / T_2	HE: 2.86 eV (434 nm)<br>LE: 2.44 eV (508 nm)	0.08 eV
T_3 (opt)	T_2 / T_3	T_1	HE: 2.35 eV (527 nm)<br>LE: 2.21 eV (562 nm)	0.31 eV

Table S5. Vertical emissions from excited-state geometries of **2**, optimized using TD-DFT

State	HE Band	LE Band	E_{calc}, in eV (λ)	$E_{T1} - E_{Tn}$ (eV)
T_1 (opt)	T_2 / T_3	T_1	HE: 3.01 eV (412 nm)<br>LE: 2.53 eV (491 nm)	--
T_2 (opt)	T_3	T_1 / T_2	HE: 2.72 eV (456 nm)<br>LE: 2.36 eV (525 nm)	0.17 eV
T_3 (opt)	T_2 / T_3	T_1	HE: 2.21 eV (561 nm)<br>LE: 2.10 eV (591 nm)	0.43 eV

6. NMR spectra

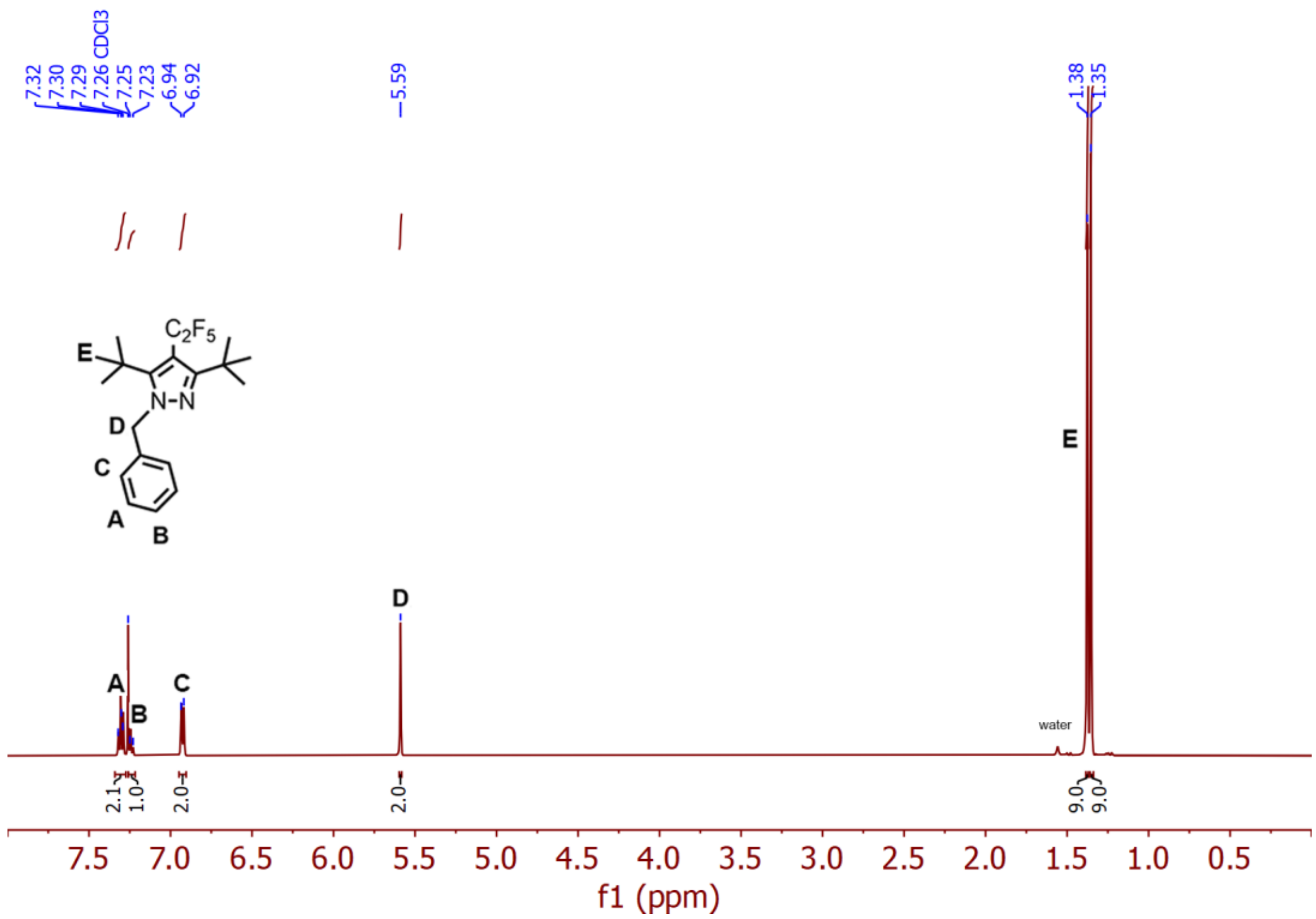

Figure S7. ^{1}H NMR spectrum of *N*-benzyl-3,5-$^{t}Bu_2$-4-C_2F_5-pyrazole (500 MHz, $CDCl_3$).

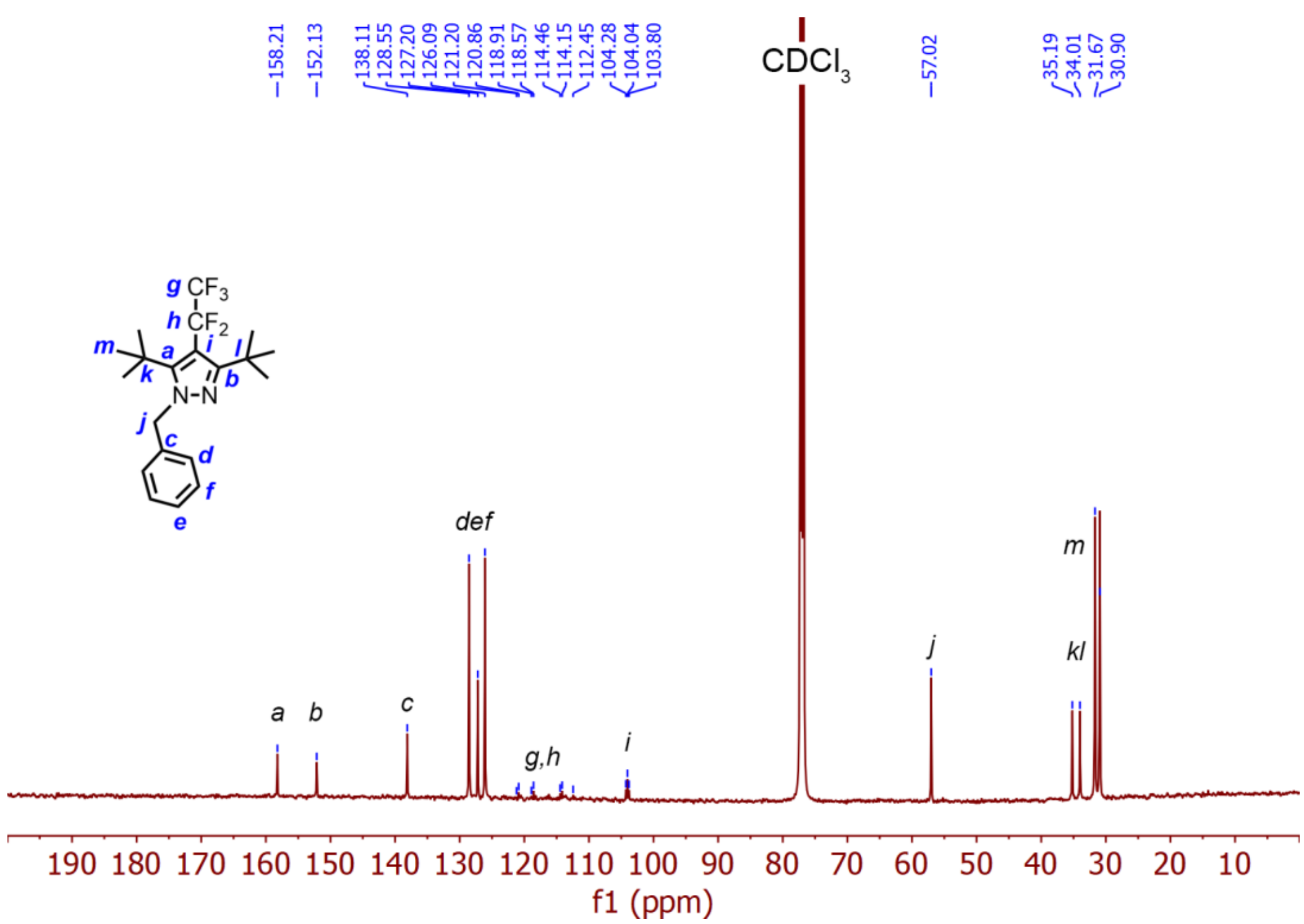

Figure S8. ^{13}C NMR spectrum of *N*-benzyl-3,5-$^{t}Bu_2$-4-C_2F_5-pyrazole (125 MHz, $CDCl_3$).

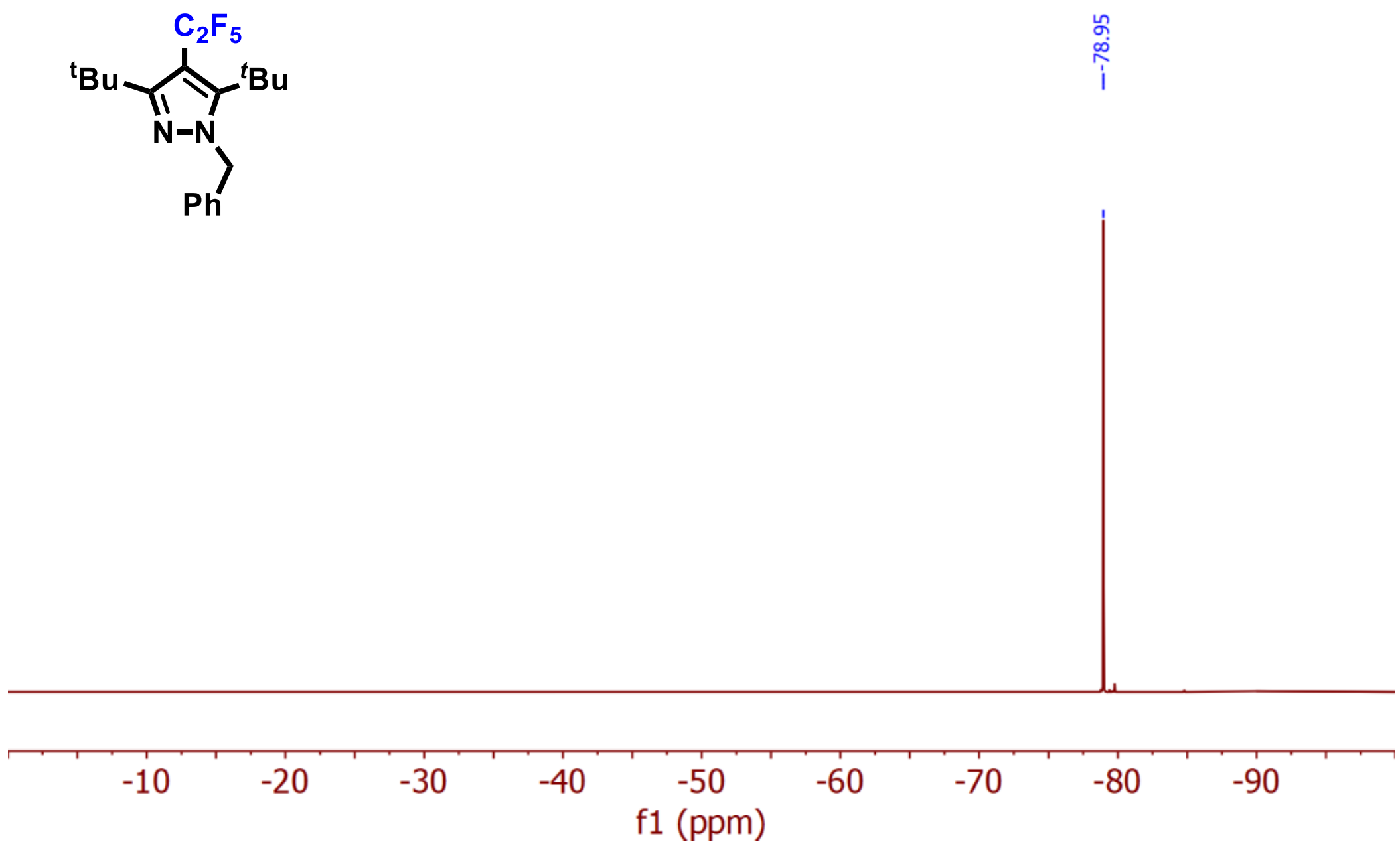

Figure S9. ^{19}F NMR spectrum of *N*-benzyl-3,5-$^{t}Bu_2$-4-C_2F_5-pyrazole (376 MHz, $CDCl_3$).

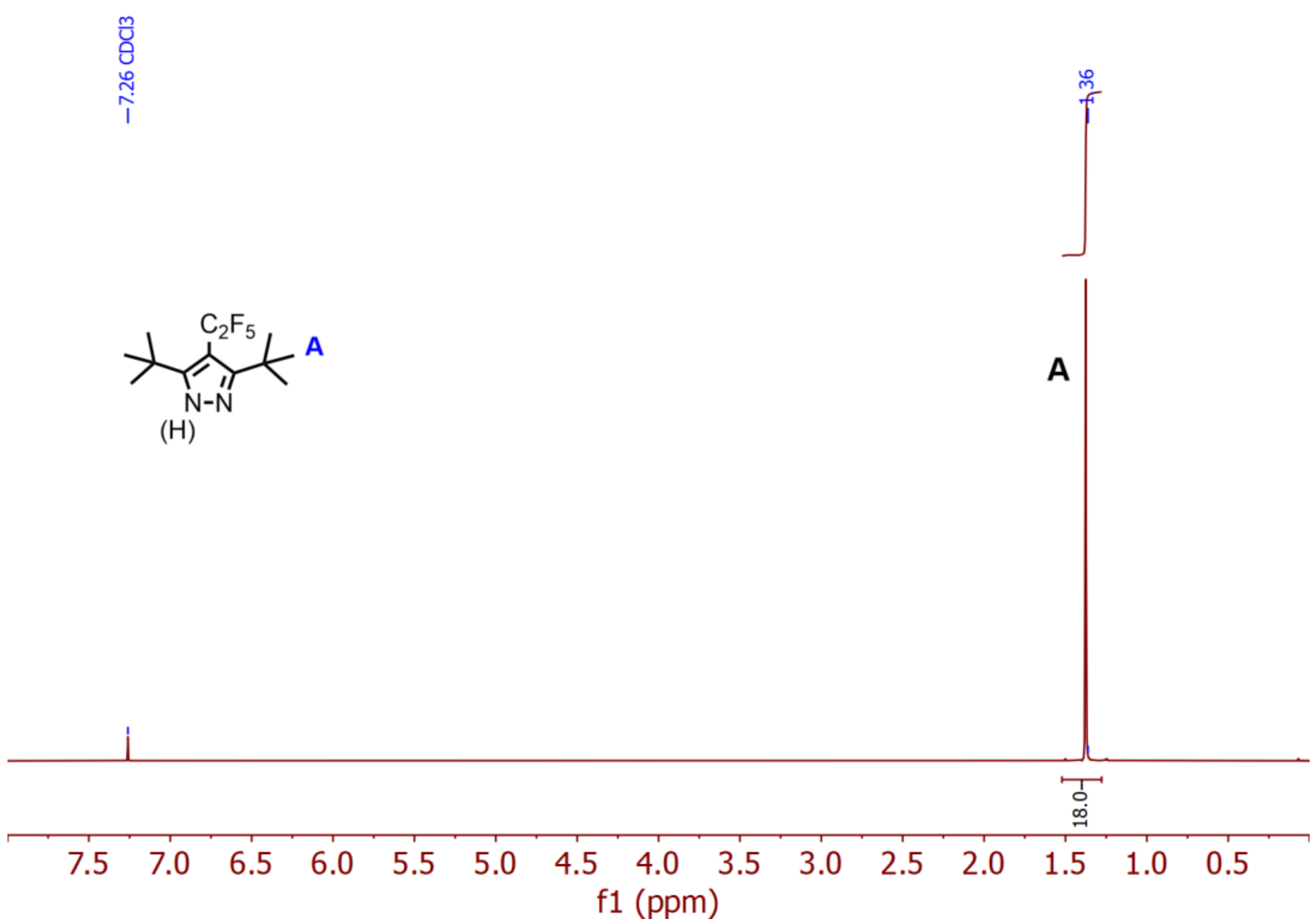

Figure S10. ^{1}H NMR spectrum of 3,5-$^{t}Bu_2$-4-C_2F_5-pyrazole **3** (500 MHz, $CDCl_3$).

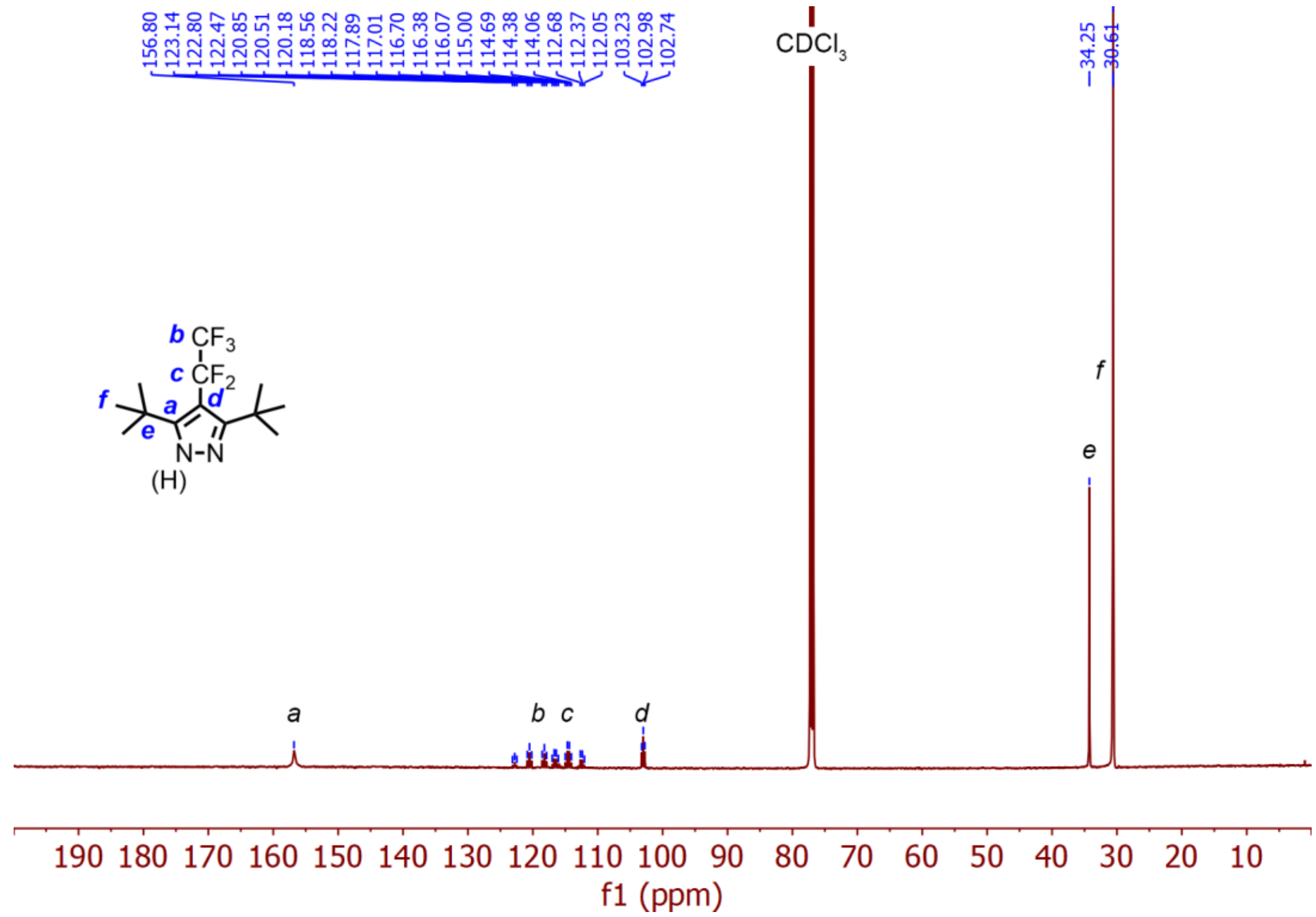

Figure S11. ^{13}C NMR spectrum of 3,5-$^{t}Bu_2$-4-C_2F_5-pyrazole **3** (125 MHz, $CDCl_3$).

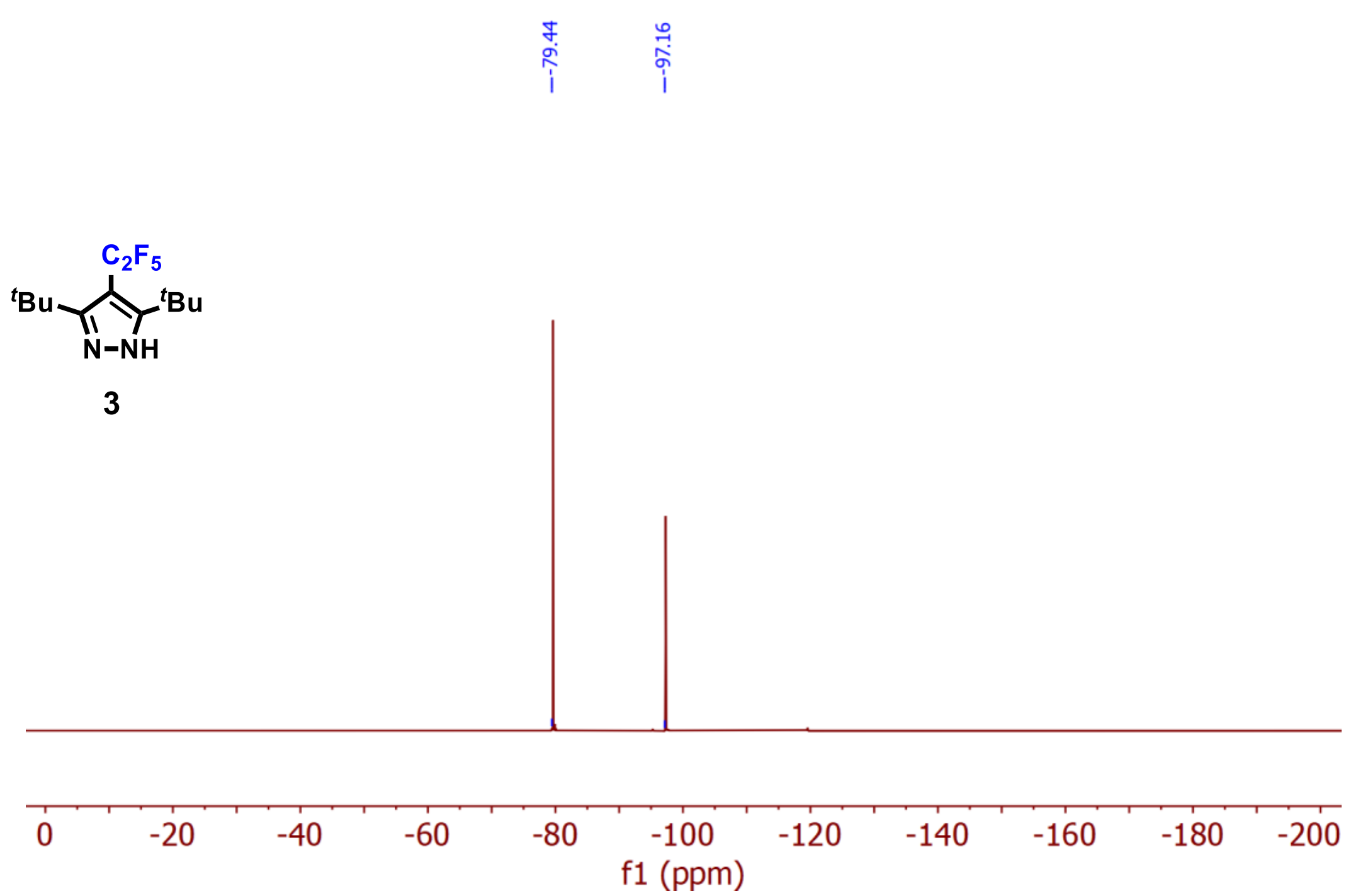

Figure S12. ^{19}F NMR spectrum of 3,5-$^{t}Bu_2$-4-C_2F_5-pyrazole **3** (376 MHz, $CDCl_3$).

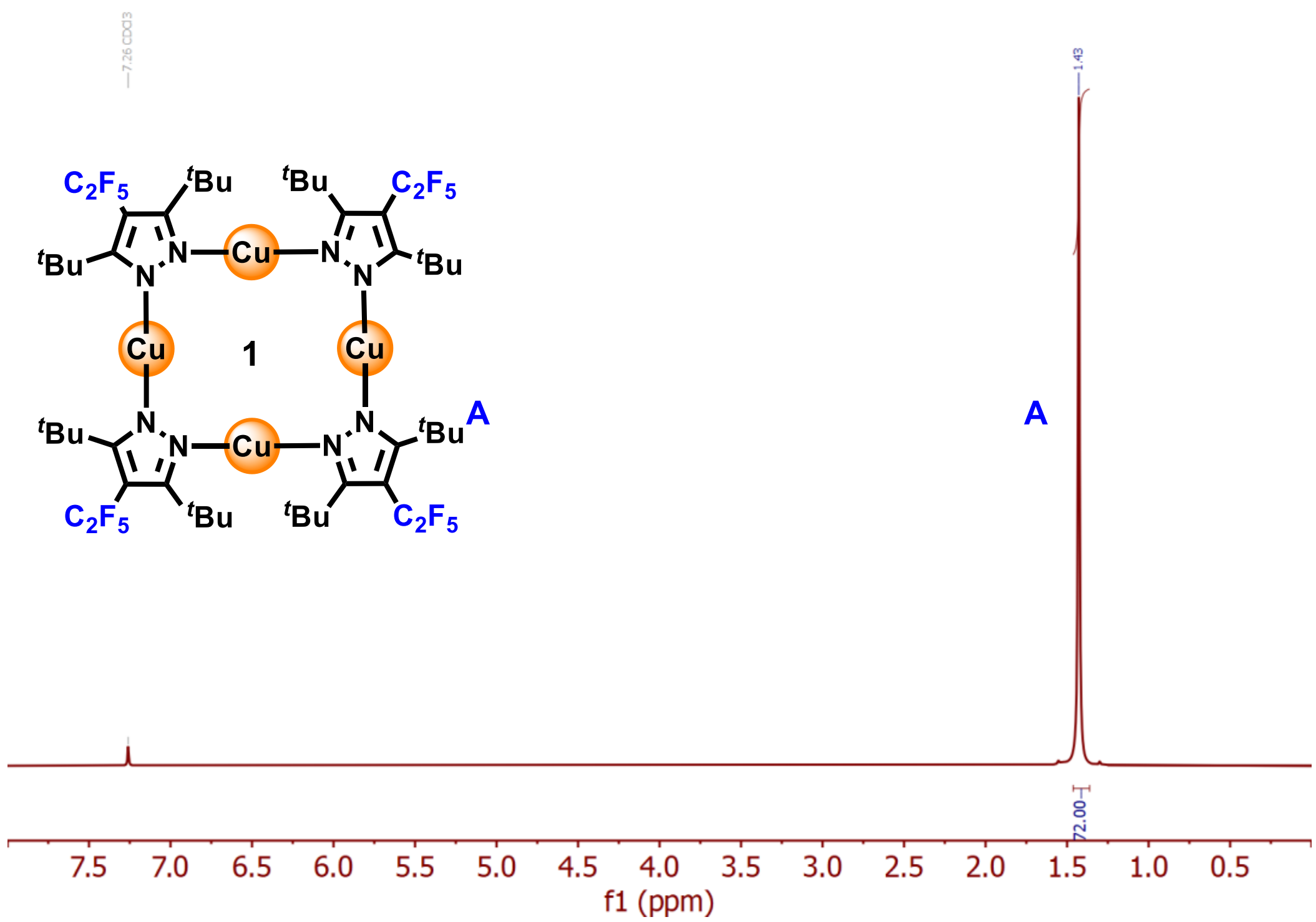

Figure S13. ^{1}H NMR spectrum of $Cu_4(C_2F_5\text{-pz})_4$ **1** (500 MHz, $CDCl_3$).

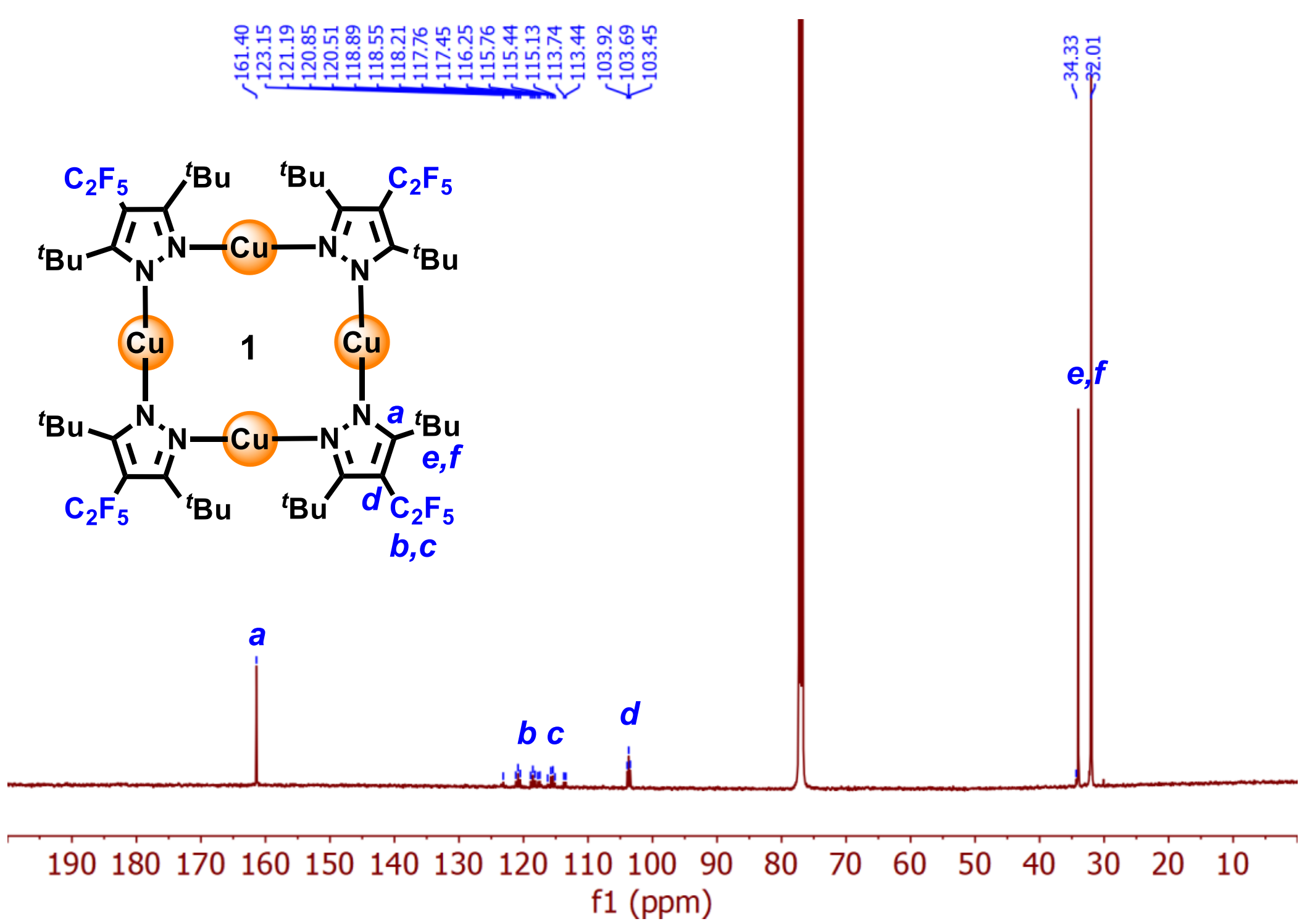

Figure S14. ^{13}C NMR spectrum of $Cu_4(C_2F_5\text{-pz})_4$ **1** (125 MHz, $CDCl_3$).

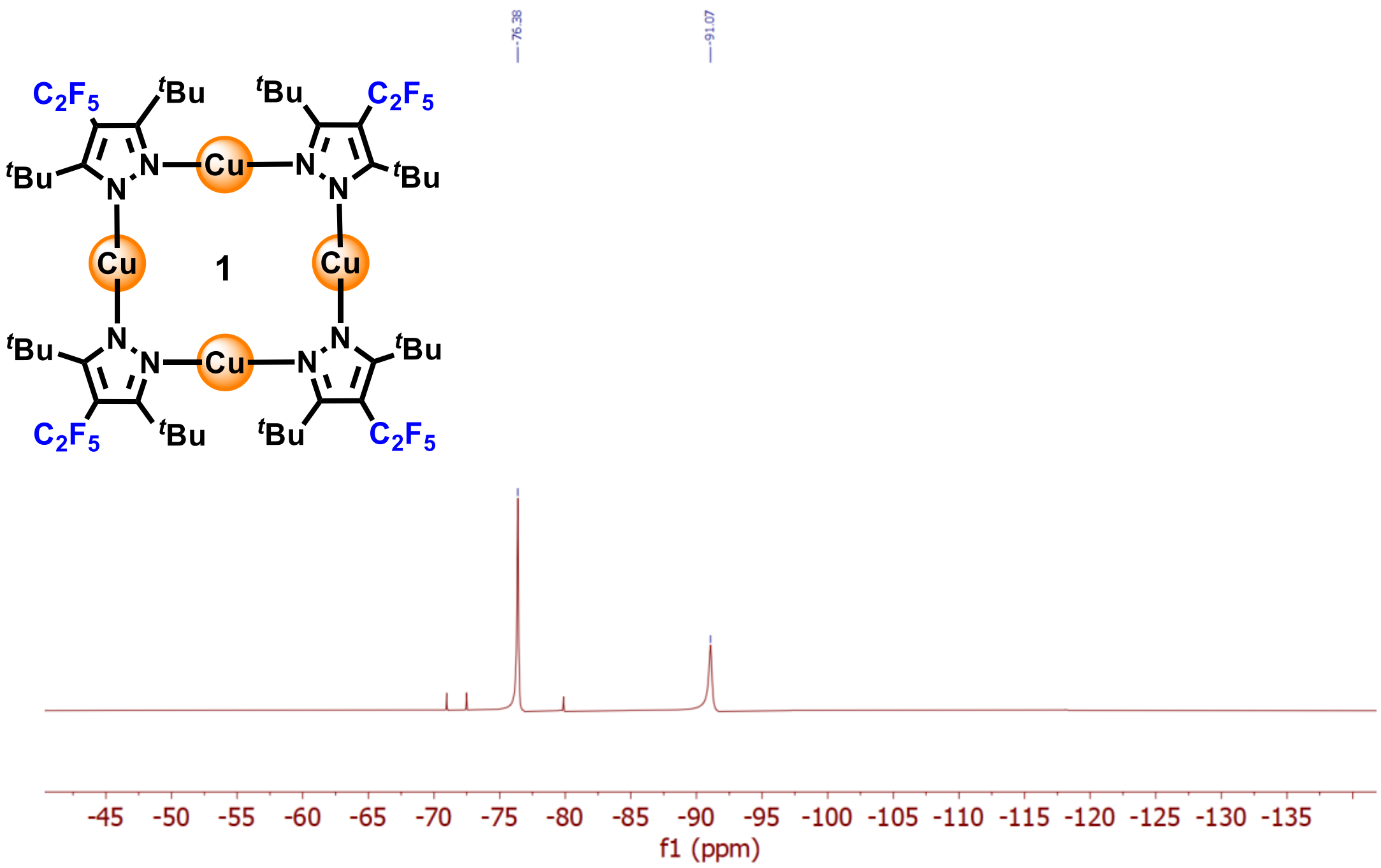

Figure S15. ^{19}F NMR spectrum of $Cu_4(C_2F_5\text{-pz})_4$ **1** (376 MHz, $CDCl_3$).

7. References

[S1] (a) F. Neese, *Wiley Interdiscip. Rev.: Comput. Mol. Sci.*, 2012, **2**, 73–78. (b) F. Neese, *Wiley Interdiscip. Rev.: Comput. Mol. Sci.*, 2025, **15**, e70019.

[S2] E. F. Pettersen, T. D. Goddard, C. C. Huang, E. C. Meng, G. S. Couch, T. I. Croll, J. H. Morris and T. E. Ferrin, *Protein Sci.,* 2021, **30**, 70–82.

[S3] (a) T. Lu and F. Chen, *J. Comput. Chem.* 2012, **33**, 580–592. (b) T. Lu, *Carbon*, 2022, **187**, 78. (c) T. Lu, *J. Chem. Phys.,* 2024, **161**, 082503.

[S4] S. K. Rajagopal, M. Zeller, S. Savikhin, L. V. Slipchenko and A. Wei, *Chem. Commun.*, 2024, **60**, 11307–11310.

[S5] J. Mestre, S. Castillón and O. Boutureira, *J. Org. Chem.*, 2019, **84**, 15087–15097.

[S6] (a) SHELXTL suite of programs, Version 6.14, 2000-2003, Bruker AXS Inc., Madison, WI, USA. (b) G. M. Sheldrick, *Acta Crystallogr A,* 2008, **64**, 112–122.

[S7] (a) G. M. Sheldrick, *Acta Crystallogr. C,* 2015, **71**, 3–8; (b) G. M. Sheldrick, Univ. Göttingen, Germany, 2018.

[S8] C. B. Hübschle, G. M. Sheldrick and B. J. Dittrich*, J. Appl. Crystallogr.*, 2011, **44**, 1281–1284.

[S9] L. Krause, R. Herbst-Irmer, G. M. Sheldrick and D. Stalke, *J. Appl. Crystallogr.*, 2015, **48**, 3–10.

[S10] D. Peng and M. Reiher, *Theor. Chem. Acc.*, 2012, **131**, 1081.

[S11] P. Pollak and F. Weigend, *J. Chem. Theory Comput.*, 2017, **13**, 3696–3705.
